# Implementation of paediatric precision oncology into clinical practice: The Individualized Therapies for Children with cancer program ‘iTHER’

**DOI:** 10.1016/j.ejca.2022.09.001

**Published:** 2022-11

**Authors:** Karin P.S. Langenberg, Michael T. Meister, Jette J. Bakhuizen, Judith M. Boer, Natasha K.A. van Eijkelenburg, Esther Hulleman, Uri Ilan, Eleonora J. Looze, Miranda P. Dierselhuis, Jasper van der Lugt, Willemijn Breunis, Linda G. Schild, Kimberley Ober, Sander R. van Hooff, Marijn A. Scheijde-Vermeulen, Laura S. Hiemcke-Jiwa, Uta E. Flucke, Mariette E.G. Kranendonk, Pieter Wesseling, Edwin Sonneveld, Simone Punt, Arjan Boltjes, Freerk van Dijk, Eugene T.P. Verwiel, Richard Volckmann, Jayne Y. Hehir-Kwa, Lennart A. Kester, Marco M.J. Koudijs, Esme Waanders, Frank C.P. Holstege, H. Josef Vormoor, Eelco W. Hoving, Max M. van Noesel, Rob Pieters, Marcel Kool, Miriam Stumpf, Mirjam Blattner-Johnson, Gnana P. Balasubramanian, Cornelis M. Van Tilburg, Barbara C. Jones, David T.W. Jones, Olaf Witt, Stefan M. Pfister, Marjolijn C.J. Jongmans, Roland P. Kuiper, Ronald R. de Krijger, Marc H.W. Wijnen, Monique L. den Boer, C. Michel Zwaan, Patrick Kemmeren, Jan Koster, Bastiaan B.J. Tops, Bianca F. Goemans, Jan J. Molenaar

**Affiliations:** a Princess Máxima Center for Pediatric Oncology, Heidelberglaan 25, 3584 CS Utrecht, the Netherlands; b Oncode Institute, Heidelberglaan 25, 3584 CS Utrecht, the Netherlands; c Department of Pathology, University Medical Center Utrecht, Heidelberglaan 100, 3584 CX Utrecht, the Netherlands; d Department of Genetics, University Medical Center Utrecht, Heidelberglaan 100, 3584 CX Utrecht, the Netherlands; e Universitäts-Kinderspital Zürich, Steinwiesstrasse 75, 8032 Zürich, Switzerland; f Department of Oncogenomics, Center for Experimental and Molecular Medicine (CEMM), Amsterdam University Medical Centers, University of Amsterdam, Meibergdreef 9, 1105 AZ Amsterdam, the Netherlands; g Center for Molecular Medicine, University Medical Center Utrecht, Heidelberglaan 100, 3584 CX Utrecht, the Netherlands; h Newcastle University, Newcastle Upon Tyne, NE1 7RU, UK; i Division Imaging & Cancer, University Medical Center Utrecht, Heidelberglaan 100, 3584 CX Utrecht, the Netherlands; j Hopp Children's Cancer Center Heidelberg (KiTZ), German Cancer Research Center (DKFZ) and German Cancer Consortium (DKTK), Im Neuenheimer Feld 280, 69120 Heidelberg, Germany; k Department of Pediatric Hematology and Oncology, Hopp Children's Cancer Center Heidelberg (KiTZ), Heidelberg University Hospital, Im Neuenheimer Feld 430, 69120 Heidelberg, Germany; l University Medical Center Utrecht, Heidelberglaan 100, 3584 CX Utrecht, the Netherlands; m Department of Pharmaceutical Sciences, Utrecht University, Universiteitsweg 99, 3584 CG Utrecht, the Netherlands

**Keywords:** Precision medicine, Molecular biology, Next-generation sequencing, Molecular targeted therapy, Hereditary, Cancer, Child, Adolescent

## Abstract

iTHER is a Dutch prospective national precision oncology program aiming to define tumour molecular profiles in children and adolescents with primary very high-risk, relapsed, or refractory paediatric tumours. Between April 2017 and April 2021, 302 samples from 253 patients were included. Comprehensive molecular profiling including low-coverage whole genome sequencing (lcWGS), whole exome sequencing (WES), RNA sequencing (RNA-seq), Affymetrix, and/or 850k methylation profiling was successfully performed for 226 samples with at least 20% tumour content. Germline pathogenic variants were identified in 16% of patients (35/219), of which 22 variants were judged causative for a cancer predisposition syndrome. At least one somatic alteration was detected in 204 (90.3%), and 185 (81.9%) were considered druggable, with clinical priority *very high* (6.1%), *high* (21.3%), *moderate* (26.0%), *intermediate* (36.1%), and *borderline* (10.5%) priority. iTHER led to revision or refinement of diagnosis in 8 patients (3.5%). Temporal heterogeneity was observed in paired samples of 15 patients, indicating the value of sequential analyses.

Of 137 patients with follow-up beyond twelve months, 21 molecularly matched treatments were applied in 19 patients (13.9%), with clinical benefit in few. Most relevant barriers to not applying targeted therapies included poor performance status, as well as limited access to drugs within clinical trial.

iTHER demonstrates the feasibility of comprehensive molecular profiling across all ages, tumour types and stages in paediatric cancers, informing of diagnostic, prognostic, and targetable alterations as well as reportable germline variants. Therefore, WES and RNA-seq is nowadays standard clinical care at the Princess Máxima Center for all children with cancer, including patients at primary diagnosis. Improved access to innovative treatments within biology-driven combination trials is required to ultimately improve survival.

## Highlights

• Implementing comprehensive molecular profiling into standard of care is feasible.
• Temporal heterogeneity is observed, indicating the value of sequential analyses.
• Molecularly matched treatments are applied in a minority of patients despite clinical benefit.
• Poor performance status & limited access to drugs within trial hamper targeted treatment.
• The multidisciplinary tumour board is crucial in translating findings into clinical decision making.

## 1 Introduction

Over the last decades, survival of paediatric oncology patients in high-income countries has significantly increased due to intense multimodal treatment strategies, improved supportive care, and centralisation of paediatric oncologic care [1,2]. However, prognosis for a subset of patients with high-risk, relapsed, and refractory cancers remains poor, and survivors are facing severe late side-effects, stressing the need for innovative treatment approaches [3].

Large-scale next-generation sequencing (NGS) studies have provided insights into the genomic landscape of paediatric cancer, which enables improving outcomes through molecularly matched treatments [[4], [5], [6]]. Multiple prospective precision-medicine programs report the feasibility of NGS in a clinical setting in a relevant timeframe and reveal a rate of actionable variants that justify the development of predictive biomarker-driven trials for childhood cancer [[7], [8], [9], [10], [11], [12]]. Large-scale programs include the international INFORM registry including thirteen countries [13], MAPPYACTS (France) [14], SM-PAEDS (UK) [15], the US-based NCI–COG Pediatric MATCH trial [16] and the Australian Zero Childhood Cancer Program [17].

The Dutch Princess Máxima Center for pediatric oncology (‘Máxima’) is Europe's largest comprehensive childhood cancer centre, registering approximately 600 new cancer cases each year in children under 18 years of age (www.skion.nl). The ‘individual Therapies’ (‘iTHER’) program is a prospective national precision oncology program, aiming to benefit children and adolescents with primary very high-risk, relapsed, or refractory paediatric tumours with the goal to evaluate feasibility and ultimately implementation into standard of care of the comprehensive molecular profiling platform. Here, we report the first results consisting of whole exome, transcriptome, and DNA methylome analysis to identify relevant somatic and germline aberrations to inform treatment. We discuss translation into treatment recommendations and explore key barriers to therapeutic application, highlighting the need for close collaboration between biologists, bioinformaticians, and clinicians.

## 2 Methods

### 2.1 Study design, patients, and sample inclusion

**Fig. 1:**
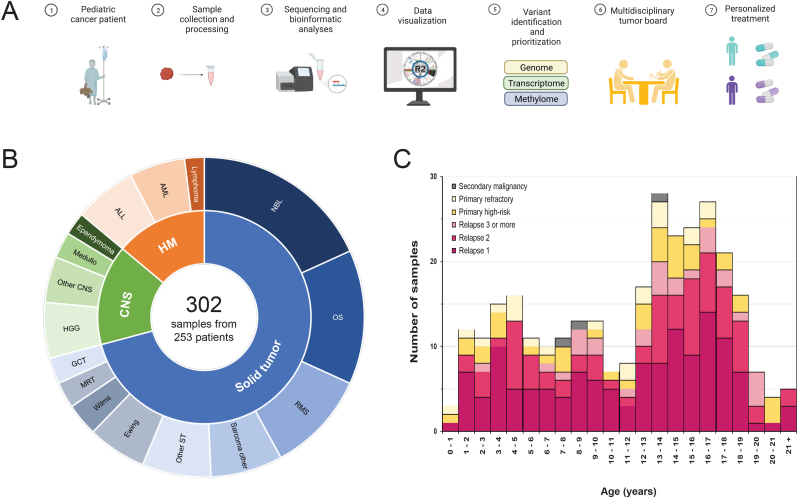
**iTHER program workflow and cohort demographics**. (**A**) Detailed iTHER pipeline is depicted. ∗ After consent, samples are processed and sequenced utilising INFORM and/or Máxima pipeline. Curated and prioritised events are discussed at the Molecular Tumour Board to identify molecularly matched treatment options. ∗ Created with BioRender.com. (**B**) iTHER cohort consisting of 302 samples from 253 patients with relapsed, refractory, or high-risk newly diagnosed paediatric cancer. The innermost ring visualises disease distribution by the three main categories: extracranial solid tumour (Solid tumour), Central Nervous System tumour (CNS) and Haematopoietic Malignancies (HM). The outer ring represents the frequency of samples within each subtype: neuroblastoma (NBL), osteosarcoma (OS), rhabdomyosarcoma (RMS), other sarcoma (Sarcoma other), other solid tumours (Other ST), Ewing sarcoma, Wilms tumour (Wilms), malignant rhabdoid tumour (MRT) and extracranial germ cell tumour (GCT); high-grade glioma (HGG), other CNS tumours (Other CNS), medulloblastoma (Medullo), Ependymoma; acute lymphoblastic leukaemia (ALL), acute myeloid leukaemia (AML), and lymphoma (Lymphoma). (**C**) Age distribution of the cohort, highlighting stage of disease: primary high-risk disease, primary refractory disease, relapse, and secondary malignancy.

The iTHER program opened after ethics approval in April of 2017 as a prospective non-interventional observational study (Netherlands Trial Register, Trial NL5728; NL56826.078.16). Fig. 1A depicts the study workflow, which was developed in close international collaboration with INFORM, sharing Standard Operating Procedures and discussing results at the international Molecular Tumour Board. Protocol details and inclusion criteria are summarised in Supplemental Table 1. iTHER was initiated at three sites in the Netherlands: the Máxima in Utrecht, Erasmus Medical Center in Rotterdam, and Amsterdam University Medical Center in Amsterdam. Since June 2018, care for all children with cancer is centralised at the Máxima and therefore iTHER continued as a single-site study. Eligible patients included children, adolescents, and young adults with (suspected) high-risk, relapsed, or refractory malignancies. After written informed consent, fresh tumour biopsy of the current disease episode, or bone marrow in case of leukaemia, as well as germline material was obtained and processed immediately to avoid delay and tissue degradation. Material was evaluated by a dedicated staff pathologist with specific expertise in paediatric oncology and distributed for routine diagnostic testing, and subsequently approved for ongoing research including iTHER, and biobanking. For solid and central nervous system (CNS) tumours included in iTHER, frozen sections were prepared to assess tumour cell percentage by haematoxylin and eosin (H&E) staining, requiring at least 20% tumour content. Clinical data including age, sex, diagnosis, administered therapy, and best response to therapy were entered into the study database.

### 2.2 Sample preparation, sequencing, data integration and visualisation

Total DNA and RNA were isolated using the AllPrep DNA/RNA/Protein Mini Kit (Qiagen) according to the manufacturer's protocol on the QiaCube (Qiagen). Comprehensive molecular profiling of somatic and germline material in the first part of this study (*n* = 141) was performed and kindly provided by the INFORM program [18,19]. In brief, data were generated utilising low-coverage Whole Genome Sequencing (lcWGS), Whole Exome Sequencing (WES), mRNA sequencing (RNA-seq), Affymetrix gene expression array and 850k Illumina EPIC DNA methylation array profiling.

Subsequent samples (*n* = 106) were sequenced at the Máxima according to the Supplemental data Extended methods, applying WES (germline and somatic), RNA-seq, and 850k Illumina Infinium EPIC DNA methylation array profiling for CNS tumours [20] or sarcomas [21]. Twenty-one samples were profiled by both centres for quality control and validation purposes.

Data were transferred to the R2 Genomics Analysis and Visualisation platform (http://r2.amc.nl).

### 2.3 Target identification, prioritisation, and reporting

Identified somatic molecular aberrations included single nucleotide variants (SNVs) and small insertions/deletions (InDels) with a variant allele fraction of >0.1; copy number variants (CNVs; defined as follows: gain logfold >1.0; amplification >2.0; deletion −1.0 or less; biallelic deletion −2.0 or less); gene fusions and other structural variants (SVs) as well as overexpressed genes (log2 Z-score >2.5 across the reference cohort) and, in selected AML patients, farnesyldiphosphate farnesyl-transferase 1 (*FDFT1*) methylation status [22]. Events were annotated based on databases, including COSMIC [23] and PeCan (http://r2.amc.nl). Of note, biallelic inactivation leading to loss-of-function of the protein was required to annotate inactivation of a tumour suppressor gene. Numerical or segmental chromosomal aberrations without single driver gene event were not reported in this study. Somatic events were classified as potentially druggable, or biologically relevant only. Results were prioritised in the context of tumour type by two independent molecular biologists with specific disease expertise, based on available (pre)clinical data and according to an algorithm previously published by INFORM (Supplemental Fig. 1) [13,24]. Germline variants in selected childhood cancer predisposition genes were classified according to ACMG guidelines (Supplemental Table 2) [25].

Following this curation process, results were discussed at the weekly multidisciplinary Molecular Tumour Board (MTB). Core members included (the treating) paediatric oncologists, early phase clinical trial oncologists, molecular pathologists, clinical geneticists, bioinformaticians, and disease-specific paediatric tumour biologists from our research department. Biologically relevant aberrations supporting and/or refining diagnosis and prognosis were reviewed, as were germline findings indicating the presence of a cancer predisposition syndrome. Specific treatment options, preferably within the context of a clinical trial, were suggested and reported in writing, but therapeutic interventions were not part of this protocol.

Clinical follow-up data were collected for all patients, captured every three months for the duration of one year (iTHER 1.0) or two years (iTHER 2.0), respectively.

## 3 Results

### 3.1 Patients and baseline characteristics

From April 2017 to April 2021, we prospectively enrolled 302 study samples derived from 253 patients in the iTHER program. Tumour categories included extracranial solid tumours (*n* = 214; 70.9%), CNS tumours (*n* = 46; 15.2%) and haematological malignancies (*n* = 42; 13.9%) (Fig. 1B). Samples were included at initial diagnosis of a high-risk tumour (*n* = 39; 12.9%), primary refractory disease (*n* = 21; 7.0%), relapsed cancer (*n* = 239; 79.1%) or at the time of diagnosis of a second malignancy (*n* = 3; 1.0%). Age at registration was nine months to 23 years (median 13.1 y; IQR 9.5 y) (Fig. 1C).

**Fig. 2:**
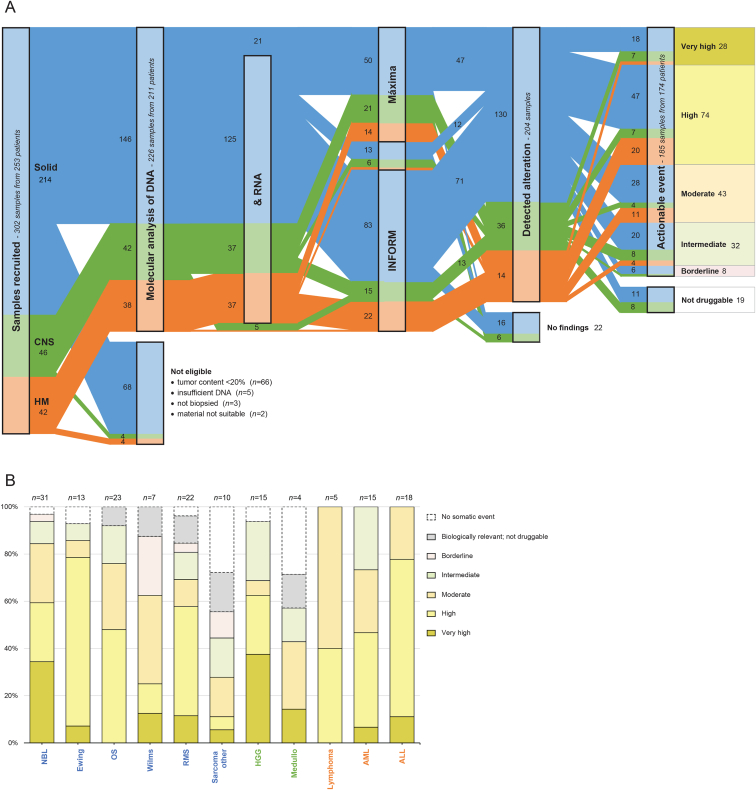
**iTHER sample flow and target priority**. (**A**) Consort flow chart for all 302 samples included. (**B**) Distribution of the highest priority level target per sample, highlighted per cancer subtype for all subgroups with 4 or more samples included: neuroblastoma (NBL), Ewing sarcoma (Ewing), osteosarcoma (OS), Wilms tumour (Wilms), rhabdomyosarcoma (RMS), other types of sarcomas (Sarcoma other), high-grade glioma (HGG) and medulloblastoma (Medullo); lymphoma (Lymphoma), acute myeloid leukaemia (AML), and acute lymphoblastic leukaemia (ALL).

The detailed cohort diagram is depicted in (Fig. 2A). Seventy-six samples were not eligible for processing, mainly due to the absence of (sufficient) tumour cells because patients were included prior to obtaining tissue and/or histological confirmation of disease recurrence. DNA and RNA were successfully isolated from 199 samples, while for 27 cases only DNA could be extracted. Molecular profiling was performed via the pipelines of INFORM (*n* = 120), the Máxima (*n* = 85), or both (*n* = 21). Data were transferred to the R2 Genomics Analysis and Visualisation platform, where a comprehensive suite of visualisations and analysis options were implemented in close collaboration with the researchers performing the molecular analysis (Supplemental Fig. 2). This has resulted in an interlinked interface where all data of a single patient can be assessed at different levels of granularity, and in addition cohort-based analyses can be readily performed. The median turnaround time from sample inclusion to MTB was similar to other programs: 30.0 working days using the INFORM pipeline and reduced to 22.5 working days after transferring to the Máxima, mainly because sample shipments were no longer required.

### 3.2 Identification and prioritisation of somatic events

**Fig. 3:**
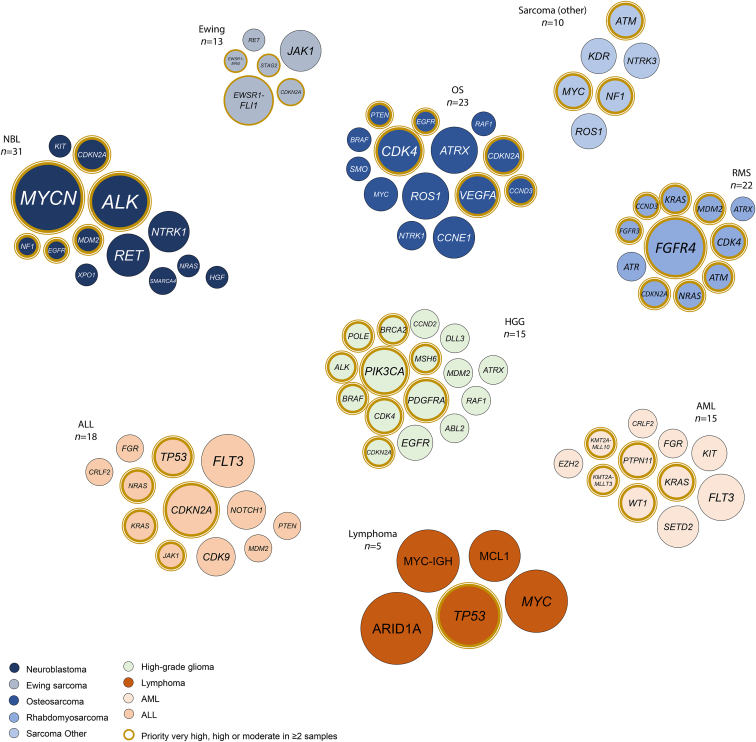
**Commonly altered druggable genes per cancer subtype**. Each bubble cloud represents a subgroup with 4 or more samples harbouring actionable events. Every bubble represents a single potentially targetable gene. Its size indicates the relative frequency within the cancer subtype. The gene is highlighted if priority score is considered *very high*, *high*, or *moderate*, and the event is observed in a minimum of two samples.

In 204 of the 226 processed samples (90.3%), at least one biologically relevant or potentially druggable somatic single gene alteration was reported (Supplemental Fig. 3A). These 737 events included SNVs/InDels (37.9%); CNVs (23.1%), overexpressed genes (25.9%), SVs/gene fusions (12.2%) as well as demethylated genes (0.9%) (Supplemental Fig. 3B). Affected pathways included kinase signalling (29.2%), DNA replication (23.7%), transcription (19.7%), epigenetic processes (10.5%), MAPK signalling (6.8%), DNA repair (2.8%), and other (7.3%) (Supplemental Fig. 3C). Nineteen samples (8.4%) had alterations that were biologically relevant but were not considered actionable in the specific tumour type. The remaining 185 samples harboured a total of 534 somatic alterations that were considered druggable, with priority levels of very high (6.1%), high (21.3%), moderate (26.0%), intermediate (36.1%) and borderline (10.5%). The distribution of the highest priority levels differs per cancer subtype and is illustrated in Fig. 2B for subgroups with four or more samples included. For example, druggable alterations occur frequently in neuroblastoma and haematological malignancies, but are less common in sarcoma and medulloblastoma. Commonly altered druggable genes per cancer subtype are shown in Fig. 3 and Supplemental Fig. 4. Genes with the highest priority for potential targeted therapy are highlighted, e.g. *ALK* in neuroblastoma and *RAS* in acute lymphoblastic leukaemia.

Results of molecular profiling revised or refined diagnosis in eight patients, including novel fusion detection by unbiased RNA-seq (Supplemental Table 3). For example, a *TPM3-NTRK1* fusion in an adolescent with a malignant peripheral nerve sheath tumour (MPNST) was identified, which is considered a very-high-priority actionable event, offering the patient a relevant treatment option (e.g. tropomyosin receptor kinase (TRK) inhibitor). As another example, a pulmonary metastatic lesion of an embryonal rhabdomyosarcoma harboured a novel *PAX3-WWTR1* fusion transcript, which was, in retrospect, also detected in the primary tumour from the shoulder [26]. Diagnosis was revised in another patient, where the histopathological diagnosis initially favoured malignant rhabdoid tumour. Inactivation of *SMARCA4* was reported due to an SNV and loss of heterozygosity (LOH). However, when looking at the broader DNA-methylation cohort, this case clustered with neuroblastomas, and the diagnosis was revised accordingly (Supplemental Fig. 5). A diagnosis of relapsed undifferentiated sarcoma was refined to *BCOR*-sarcoma due to the identification of the BCOR-CCNB3 fusion and absent EWSR1 fusion. One patient with suspected relapsed ATRT was classified as diffuse paediatric-type high-grade glioma, H3-wildtype and IDH-wildtype (pedRTK1b subtype), and thus a second primary tumour, allowing for an adapted treatment strategy.

Two independent samples were submitted for molecular analysis in 15 patients (Supplemental Fig. 6A). Except for P07-aRMS, all were obtained at consecutive timepoints. Temporal heterogeneity was observed in all tumour types in this small subset, underscoring the previously demonstrated value of sequential analysis in paediatric cancer [13,27,28]. Of three patients (P07-aRMS (*n* = 1) and neuroblastoma (NBL, *n* = 2)), primary tumour and metastatic site were profiled (Supplemental Fig. 6B). Samples of P07-aRMS were obtained at the same timepoint but showed different events: the primary tumour exhibited an *ATRX*-deletion, whilst the metastasis harboured SNVs in *ATR* and *RAF1*, a biallelic *BCOR*-deletion, and a *MYCN*-amplification. The NBL samples were sequenced at different time points, revealing novel molecular information. For example, in tumour P12-NBL, *RAS*-*MAPK* activation in the primary tumour occurred through loss of *NF1*, whilst it occurred in the metastatic lesion due to *BRAF*-mutation. In osteosarcoma (*n* = 2), results also differed between subsequent metastatic samples, possibly reflecting the complex karyotype, intratumor heterogeneity, and/or tumour evolution. Finally, fusion gene transcripts were retained over all samples of a given sarcoma patient.

### 3.3 Germline cancer predisposing variants

**Fig. 4:**
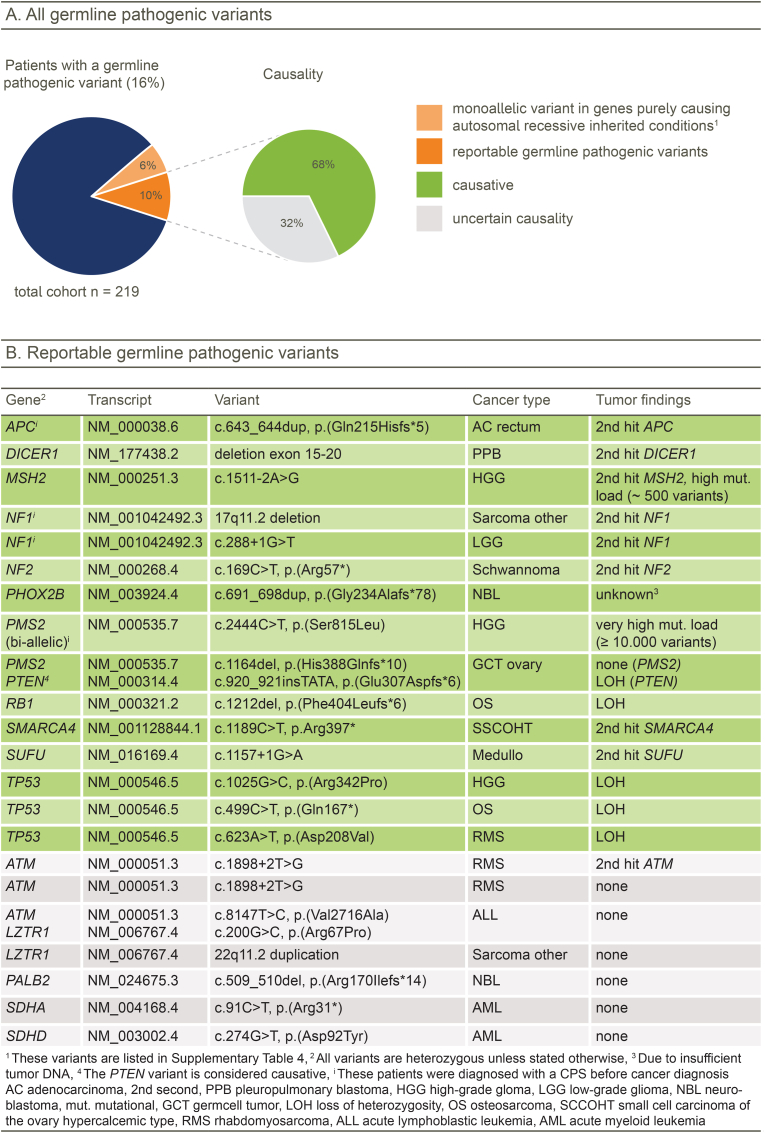
**Germline pathogenic variants**. (**A**) All identified germline pathogenic variants in the cohort with a subset of reportable cancer-associated germline variants. (**B**) Reportable germline pathogenic variants in detail.

Germline pathogenic variants were identified in 35/219 (16%) of patients for whom germline sequence results were available (Fig. 4A and Supplemental Table 4). In 22 patients (10%) the detected pathogenic variant was judged causative for a cancer predisposition syndrome (CPS) and was thus reported to the patient after clinical validation. Variants were found across 17 genes including *ATM* (*n* = 3) and *TP53* (*n* = 3). All reported pathogenic variants were heterozygous, except for a homozygous *PMS2* pathogenic variant. Two patients were found to carry a large deletion affecting (part of) a cancer predisposing gene (i.e. *DICER1* and *NF1*). The germline pathogenic variant was known prior to participating in this study in eight out of these 22 children (36%). In 15 patients (6.8%), the germline pathogenic variant contributed to the development of patient's cancer (Fig. 4B). In 14 of these 15 patients, corresponding molecular features were identified in the tumour, including a second hit in the gene affected by a germline pathogenic variant in eight tumours, LOH at the locus in five tumours, and a high mutational load in two tumours with a germline mismatch repair defect (including one with a somatic second hit). In seven patients we identified pathogenic variants in genes that, based on the current available literature, have not been associated with the type of cancer in these children yet. In all but one case, the variants were still reported because of risk for later-onset cancers, such as breast cancer in *ATM* and *PALB2* mutation carriers. These adult-onset CPS genes are included in our gene panel because biallelic variants cause paediatric-onset CPSs. For several adult CPS genes, sequencing studies revealed a possible enrichment of heterozygous pathogenic germline variants in children with cancer, although the causality of these variants in childhood cancer still has to be explored for most cancer types [4,7,17,29].

### 3.4 Molecularly matched treatment and clinical follow up

**Fig. 5:**
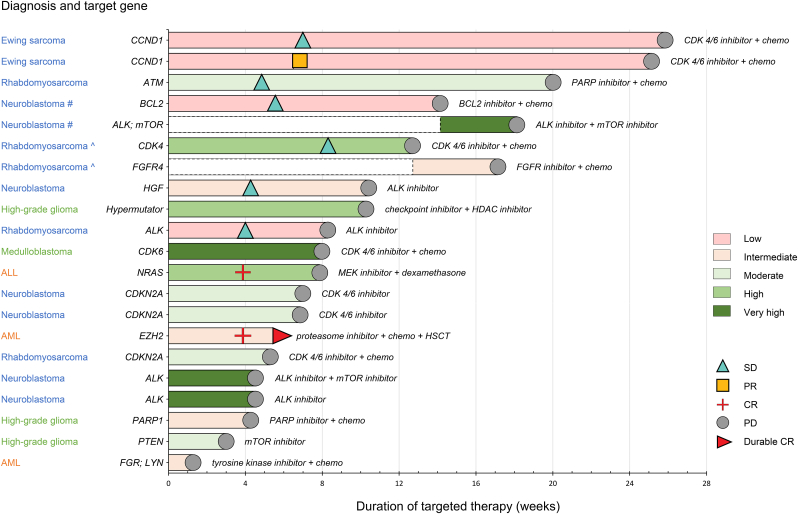
**Applied molecularly matched treatments and clinical response**. Twenty-one molecularly matched therapies were initiated in 19 patients. Time on treatment is depicted as per the prioritised target. Of note, 2 patients received 2 consecutive treatments each: one patient with neuroblastoma (#) and one patient with rhabdomyosarcoma (ˆ). x axis is time on treatment (weeks). The colour of the bars indicates the priority score of the targeted alteration. Responses are indicated by the symbols. (For interpretation of the references to colour in this figure legend, the reader is referred to the Web version of this article).

Clinical follow up, including received therapy and treatment response, was obtained for all patients. At the time of data census, 137 patients included for molecular analysis completed a minimum of 12 months of clinical follow-up. Twenty-one molecularly matched treatments were applied in 19 patients (13.9%), targeting *very high* (*n* = 4), *high* (*n* = 3), *moderate* (*n* = 5), and *intermediate* or *lower* (*n* = 9) priority events (Fig. 5 and Supplementary Table 5). Combined treatments were applied in 71.4%, consisting of a targeted agent and chemotherapy (*n* = 12) or two targeted agents (*n* = 3). Eleven treatments were administered within the context of a clinical trial (52.4% of the 21 treatments). One patient with relapsed refractory AML and a nonsense mutation in *EZH2* (D725∗) of intermediate priority, treated with the proteasome inhibitor bortezomib in combination with cytarabine, achieved complete remission followed by allogeneic stem cell transplantation (HSCT) and remains in remission after four years of follow up. Two patients diagnosed with relapsed Ewing sarcoma and suggested cell cycle activation due to high expression of *CCND1* (expression log2 Z-score 2.2 and 2.0, respectively; low priority) remained on a regimen of ribociclib combined with topotecan and temozolomide for 181 and 176 days, respectively, as reported by others [30]. Mean overall survival in the cohort was 120 days after inclusion in the iTHER program, reflecting the poor-prognosis subgroups.

### 3.5 Barriers to applying targeted treatment

Despite a high number of actionable events, matched molecular treatments were applied in only a small subset of patients. To gain a deeper understanding of hurdles to allocation of molecularly matched drugs, we performed individual surveys with 27 physicians and subsequent one-on-one interviews with 17 clinicians, resulting in the evaluation of 53 targets of very high, high, and moderate priority. In this selected subgroup, molecularly matched therapy was applied in 24% of the evaluated targets (Supplemental Fig. 7A), of which 18% were scored very high priority, 46% high and 36% moderate (Supplemental Fig. 7B). The most common reasons for not starting a patient on a matched therapy were related to the clinical status of the patient: either the disease remained stable on (palliative) conventional treatment (28.3%), or performance status was too poor (24.5%). In other cases, the patient and/or their family declined (18.9%), or there was lack of access to the drug, either within the context of a clinical trial or off-label (17.0%) (Supplemental Fig. 7C).

## 4 Discussion

Here we present the successful establishment of the prospective precision medicine program iTHER in the Netherlands for children and adolescents with cancer. The program has demonstrated significant value of WES, RNAseq and DNA methylome profiling by detecting pathogenic variants in 90.3% of samples within a clinically relevant timeframe, matching previously published studies [7,8,10,11,13,17,19,28,[31], [32], [33], [34]]. In addition, sequential analysis detected temporal heterogeneity, as published by others [13,27,28]. The frequency of reportable germline pathogenic variants in our cohort (10%) is consistent with most other studies but lower than observed in a cohort of high-risk paediatric cancer patients in Australia (16.2%) [4,7,17,29]. However, these studies are not directly comparable because of different inclusion criteria and gene panels used. Consequently, at our center, WES and RNA-seq, as well as DNA methylome profiling for CNS tumours and sarcoma, are now standard of care for all children with newly diagnosed, relapsed or refractory tumours to facilitate precision diagnostics and improve cure rates.

Unbiased RNA-seq in our cohort did not only contribute to identifying clinically relevant gene fusions and gene expression targets, but also refined diagnosis in a subset of patients, as published before [35]. However, it remains possible that clinically relevant genetic alterations have been missed in our study due to the limitations of the techniques applied. As reported by others, utilising WGS might improve the identification of mutational signatures (e.g. deficiency of homologous recombination (HR)-mediated double strand break repair and sensitivity to poly(ADP-ribose) polymerase (PARP) inhibitors [36]), activated oncogenes through enhancer hijacking (e.g. Telomerase Reverse Transcriptase (TERT) in neuroblastoma [37] or *growth factor independent 1* family protooncogenes *GFI1* and *GFI1B* and PR domain-containing protein 6 (PRDM6) in medulloblastoma [38,39]), as well as microdeletions [17,40]. The added value of implementing combined WGS and transcriptome sequencing in a clinical setting is currently being studied in our center. However, this won't be feasible in all centers, and one might consider limited panel sequencing at relapse only to select patients for ongoing clinical trials.

Definition and prioritisation of actionable events have not been standardised and harmonised in paediatric oncology, as opposed to adult oncology (e.g. NCI-MATCH Tier or The European Society for Medical Oncology (ESMO) Scale for Clinical Actionability of molecular Targets (ESCAT)) [41]. Paediatric studies are difficult to compare due to wide variability in reported cohorts and NGS pipelines. For example, in solid tumours and CNS tumours within iTHER, inactivation of *TP53* was not considered actionable, consistent with the INFORM and ZERO programs [17,19]. However, in ALL and non-Hodgkin lymphoma within iTHER, *TP53* mutation was considered potentially druggable with a moderate priority score based on early phase clinical trials with APR-246 [42,43]. *TP53* alterations were also considered investigational in MAPPYACTS [14]. As another example, gene expression outliers are considered relevant with a log2 Z-score +2.5 relative to all reference samples, independent of any genetic alteration. However, there is no consensus on this definition or the priority of overexpressed genes as a biomarker. Whether a gene is identified not only depends on pathway activation, but also on the composition of the cohort. One might consider a gene overexpressed (i.e. a potential biomarker), if the expression is high compared to other tumours of the same (sub) type. For example, in two patients with prolonged stable disease on ribociclib combined with chemotherapy, *CCND1* was not overexpressed compared with the total cohort but highly overexpressed with other Ewing sarcomas [30]. Given these complexities in result interpretation, the expertise of the multidisciplinary MTB has been crucial to translate molecular profiles into potential clinical benefit for our patients [44].

Genomic and clinical data sharing is limited thus far due to multiple factors, including privacy regulations. Multiple platforms aim to analyse and publicly visualise genomic data, since effective data sharing is key to accelerating research. We used the R2 Genomics Analysis and Visualisation Platform to analyse and visualise molecular and clinical metadata, and cohort-based analyses can be readily performed. This web-based application is extensively used by the paediatric community to integrate genomic and clinical data as well as *in vitro* and *in vivo* model systems and drug sensitivity profiles, including the ‘Innovative Therapies for Children with Cancer Pediatric Preclinical Proof-of-concept Platform’ (ITCC-P4; https://www.itccp4.eu/). To explore alterations, we also used the St. Jude PECAN application, an expanding cloud-based data-sharing ecosystem with genomic data from >10,000 paediatric patients including disease-specific Cosmic and ClinVar annotated variants (https://www.stjude.cloud). Both bioinformatic platforms have their specific qualities and can be used in a complimentary manner. Comprehensive genomic and transcriptomic analysis to inform therapy assignment has clinical benefit in a subset of patients. INFORM recently demonstrated that patients with a *very high* priority level target and molecularly matched treatment had a significantly longer Progression Free Survival [19,45]. The Zero Childhood Cancer Program also published favourable outcomes of targeted treatment compared with unselected phase I clinical trials. Remarkably, the clinical outcome did not correlate with the tier score of the recommended targeted agent, similar to the iTHER cohort, as depicted in Fig. 5 [17]. Although other studies such as iCAT [8] report a lack of clinical response, results should be interpreted with caution due to the heterogeneous character of the cohorts and methods, again stressing the need for harmonisation and collaboration. Although many targetable events were identified, only a minority of patients received targeted treatment. As in other studies, a declining clinical condition was one of the main reasons not to include a patient in a clinical trial or off-label treatment. Pediatric MATCH reported poor clinical status as a factor in prohibiting enrolment on a treatment protocol in 16% of patients [46]. Hence, molecular profiling should be considered at an earlier disease stage, identifying patients that might benefit from targeted treatment, as demonstrated recently across all stages [28]. In addition, matched treatment is hampered by limited access to biomarker-driven trials with combination strategies, for example ESMART (NCT02813135) and INFORM2 (NCT03838042). Availability of approved molecular targeted drugs for paediatric patients is still limited compared with adult indications and many new targeted drugs lack dosage guidelines and efficacy data in children. Targeted therapy development is complicated by the fact that paediatric malignancies show a relative paucity of targetable mutations as well as distinct molecular alterations compared to adult cancers, suggesting new therapeutic agents are required for paediatric cancer. In addition, there is a lack of available clinical trials and a small number of eligible patients for each study since paediatric cancer remains a rare disease [12]. Several multi-stakeholder paediatric platforms, for example ACCELERATE (https://www.accelerate-platform.org/), aim to implement innovative strategies supported by preclinical testing as proposed by collaborative groups including ITCC-P4, supported by a changing regulatory environment [47,48]. This will include studying spatio-temporal genetic heterogeneity and clonal evolution in specific subgroups and utility of novel methods including *in vitro* drug sensitivity, single-cell RNA-seq and sequencing circulating tumour DNA to guide clinical decision making in the future.

## 5 Conclusion

The iTHER program of the Princess Máxima Center demonstrates the establishment of a successful precision medicine program across all ages and tumour types in paediatric oncology that identifies diagnostic, prognostic, and targetable alterations as well as reportable germline variants within a clinically relevant timeframe. Nowadays, all children with newly diagnosed, relapsed or refractory tumours are offered WES and RNA-seq in our center as standard of care, complemented by DNA-methylation for CNS tumours and sarcomas, to facilitate precision diagnostics and improve cure rates. Standardisation of data analysis and target prioritisation as well as improved access to targeted treatments within combination trials are required to translate findings from precision medicine programs into clinical care and eventually improve survival.

## Author contributions

**Karin P.S. Langenberg**: Writing - original draft, Project administration, Conceptualisation, Data curation, Formal analysis, Investigation, Methodology, Validation, Visualisation, Writing - review & editing; **Michael T. Meister**: Data curation, Formal analysis, Investigation, Writing - review & editing, Methodology; **Jette J. Bakhuizen**: Data curation, Formal analysis, Investigation, Writing - original draft, Writing - review & editing, Methodology; **Judith M. Boer**: Data curation, Formal analysis, Investigation, Writing - review & editing, Methodology; **Natasha K.A. van Eijkelenburg**: Investigation, Writing - review & editing; **Esther Hulleman**: Data curation, Formal analysis, Investigation, Writing - review & editing; **Uri Ilan**: Data curation, Writing - review & editing, Formal analysis; **Eleonora J. Looze**: Data curation, Formal analysis, Investigation, Writing - review & editing; **Miranda P. Dierselhuis**: Formal analysis; **Jasper van der Lugt**: Formal analysis, Writing - review & editing; **Willemijn Breunis**: Formal analysis; **Linda G. Schild**: Investigation, Project administration; **Kimberley Ober**: Investigation, Project administration; **Sander R. van Hooff**: Data curation, Writing - review & editing; **Marijn A. Scheijde-Vermeulen**: Methodology, Writing - review & editing; **Laura S. Hiemcke-Jiwa**: Methodology, Writing - review & editing; **Uta E. Flucke**: Methodology, Writing - review & editing; **Mariette E.G. Kranendonk**: Methodology, Writing - review & editing; **Pieter Wesseling**: Methodology, Writing - review & editing; **Edwin Sonneveld**: Methodology, Writing - review & editing; **Simone Punt**: Formal analysis, Writing - review & editing; **Arjan Boltjes**: Software; **Freerk van Dijk**: Methodology, Software, Investigation, Writing - review & editing; **Eugene T.P. Verwiel**: Software; **Richard Volckmann**: Methodology, Software; **Jayne Y. Hehir-Kwa**: Methodology, Software, Data curation, Formal analysis, Writing - review & editing; **Lennart A. Kester**: Methodology, Data curation, Formal analysis, Writing - review & editing; **Marco M.J. Koudijs**: Data curation, Formal analysis, Writing - review & editing; **Esme Waanders**: Data curation, Formal analysis, Writing - review & editing; **Frank C.P. Holstege**: Supervision, Funding acquisition, Methodology, Resources; **H. Josef Vormoor**: Writing - review & editing; **Eelco W. Hoving**: Writing - review & editing; **Max M. van Noesel**: Writing - review & editing; **Rob Pieters**: Writing - review & editing; **Marcel Kool**: Data curation, Formal analysis, Writing - review & editing; **Miriam Stumpf**: Project administration; **Mirjam Blattner-Johnson**: Data curation, Formal analysis; **Gnana P. Balasubramanian**: Data curation, Formal analysis; **Cornelis M. Van Tilburg**: Writing - review & editing; **Barbara C. Jones**: Formal analysis, Writing - review & editing; **David T.W. Jones**: Resources, Writing - review & editing; **Olaf Witt**: Writing - review & editing; Resources; **Stefan M. Pfister**: Writing - review & editing; Resources; **Marjolijn C.J. Jongmans**: Data curation, Formal analysis, Funding acquisition, Supervision, Writing - review & editing; **Roland P. Kuiper**: Data curation, Formal analysis, Funding acquisition, Supervision, Writing - review & editing; **Ronald R. de Krijger**: Data curation, Formal analysis, Methodology, Resources, Writing - review & editing; **Marc H.W. Wijnen**: Resources, Supervision, Writing - review & editing; **Monique L. den Boer**: Formal analysis, Methodology, Supervision; **C. Michel Zwaan**: Funding acquisition, Formal analysis, Methodology, Supervision; **Patrick Kemmeren**: Funding acquisition, Resources, Methodology, Software, Data curation, Formal analysis, Writing - review & editing; **Jan Koster**: Funding acquisition, Resources, Methodology, Software, Data curation, Formal analysis, Visualisation, Writing - review & editing; **Bastiaan B.J. Tops**: Funding acquisition, Resources, Methodology, Data curation, Supervision, Writing - review & editing; **Bianca F. Goemans**: Funding acquisition, Conceptualisation, Investigation, Supervision, Writing - review & editing; **Jan J. Molenaar**: Conceptualisation, Methodology, Writing - review & editing, Supervision, Project administration, Funding acquisition.

## Funding

We are grateful to all funders.

The iTHER study was supported by grants from 10.13039/501100001826ZonMw (project number 848101004) and the Dutch Foundation Children Cancer-free (KiKa Core funding). This work is part of the research program Vernieuwingsimpuls Vidi (Combining targeted compounds in neuroblastoma tumours; is two better than one?) with project number 91716482, which is partly financed by the Dutch Research Council (NWO). This project also received funding from the 10.13039/501100000781European Research Council (ERC) under the European Union's Horizon 2020 research and innovation program, grant agreement No 716079 Predict, as well as grant agreement No 826121 iPC. Jette Bakhuizen, Freerk van Dijk, Roland Kuiper and Marjolijn Jongmans were supported by a grant from the Dutch Foundation Children Cancer-free (KiKa, project number 355).

The INFORM program is financially supported by the 10.13039/100008658German Cancer Research Center (DKFZ), the German Cancer Consortium (DKTK), the German Federal Ministry of Education and Research (BMBF), the German Federal Ministry of Health (BMG), the Ministry of Science, Research and the Arts of the State of Baden-Württemberg (MWK BW); the German Cancer Aid (DKH), the German Childhood Cancer Foundation (DKS), RTL television, the aid organisation BILD hilft e.V. (Ein Herz für Kinder) and the generous private donation of the Scheu family.

## Data availability

WES, RNA-sequencing, and DNA methylation data generated by this study are available from the European Genome Archive, accession number XX (submitted).

## Conflict of interest statement

The authors declare that they have no known competing financial interests or personal relationships that could have appeared to influence the work reported in this paper.
