# Corrigendum to “Implementation of paediatric precision oncology into clinical practice: The individualized therapies for children with cancer program ‘iTHER’” [Eur J Cancer 175 (2022) 311–325]

**DOI:** 10.1016/j.ejca.2025.115423

**Published:** 2025-05-15

**Authors:** Karin P.S. Langenberg, Michael T. Meister, Jette J. Bakhuizen, Judith M. Boer, Natasha K.A. van Eijkelenburg, Esther Hulleman, Uri Ilan, Eleonora J. Looze, Miranda P. Dierselhuis, Jasper van der Lugt, Willemijn Breunis, Linda G. Schild, Kimberley Ober, Sander R. van Hooff, Marijn A. Scheijde-Vermeulen, Laura S. Hiemcke-Jiwa, Uta E. Flucke, Mariette E.G. Kranendonk, Pieter Wesseling, Edwin Sonneveld, Simone Punt, Arjan Boltjes, Freerk van Dijk, Eugene T.P. Verwiel, Richard Volckmann, Jayne Y. Hehir-Kwa, Lennart A. Kester, Marco M.J. Koudijs, Esme Waanders, Frank C.P. Holstege, H. Josef Vormoor, Eelco W. Hoving, Max M. van Noesel, Rob Pieters, Marcel Kool, Miriam Stumpf, Mirjam Blattner-Johnson, Gnana P. Balasubramanian, Cornelis M. Van Tilburg, Barbara C. Jones, David T.W. Jones, Olaf Witt, Stefan M. Pfister, Marjolijn C.J. Jongmans, Roland P. Kuiper, Ronald R. de Krijger, Marc H.W. Wijnen, Monique L. den Boer, C. Michel Zwaan, Patrick Kemmeren, Jan Koster, Bastiaan B.J. Tops, Bianca F. Goemans, Jan J. Molenaar

**Affiliations:** a Princess Máxima Center for Pediatric Oncology, Heidelberglaan 25, Utrecht 3584 CS, the Netherlands; b Oncode Institute, Heidelberglaan 25, Utrecht 3584 CS, the Netherlands; c Department of Pathology, University Medical Center Utrecht, Heidelberglaan 100, Utrecht 3584 CX, the Netherlands; d Department of Genetics, University Medical Center Utrecht, Heidelberglaan 100, Utrecht 3584 CX, the Netherlands; e University Medical Center Utrecht, Heidelberglaan 100, Utrecht 3584 CX, the Netherlands; f Department of Oncogenomics, Center for Experimental and Molecular Medicine (CEMM), Amsterdam University Medical Centers, University of Amsterdam, Meibergdreef 9, Amsterdam 1105 AZ, the Netherlands; g Center for Molecular Medicine, University Medical Center Utrecht, Heidelberglaan 100, Utrecht 3584 CX, the Netherlands; h Newcastle University, Newcastle Upon Tyne NE1 7RU, UK; i Division Imaging & Cancer, University Medical Center Utrecht, Heidelberglaan 100, Utrecht 3584 CX, the Netherlands; j Hopp Children's Cancer Center Heidelberg (KiTZ), German Cancer Research Center (DKFZ) and German Cancer Consortium (DKTK), Im Neuenheimer Feld, Heidelberg 69120, Germany; k Department of Pediatric Hematology and Oncology, Hopp Children's Cancer Center Heidelberg (KiTZ), Heidelberg University Hospital, Im Neuenheimer Feld 430, Heidelberg 69120, Germany; l Department of Pharmaceutical Sciences, Utrecht University, Universiteitsweg 99, Utrecht 3584 CG, the Netherlands; m Universitäts-Kinderspital Zürich, Steinwiesstrasse 75, Zürich 8032, Switzerland

The authors regret the delay of EGA access numbers. WES, RNA-sequencing, and DNA methylation data generated by this study are available from the European Genome Archive, accession numbers EGAD00001010178 and EGAD00001010179.

The authors would like to apologise for any inconvenience caused.

