# Supplementary material for: Implementation of paediatric precision oncology into clinical practice: The Individualized Therapies for Children with cancer program ‘iTHER’
Source: Eur J Cancer. 2022 Nov;175:311–25. doi: 10.1016/j.ejca.2022.09.001 (PMC9586161; doi:10.1016/j.ejca.2022.09.001)
Supplement: Multimedia component 1 [file mmc1.docx]

# ­SUPPLEMENTAL FILES_LEGEND

**Supplemental Figure 1. Prioritization of somatic events on a 7-scale algorithm, as published by INFORM**

https://doi.org/10.1016/j.ejca.2016.06.009

**Supplemental Figure 2. R2 Genomics Analysis and Visualization platform**

http://r2.amc.nl

**Supplemental Figure 3. Molecular findings within the iTHER cohort.**

(**a**) Oncoprint of all detected somatic aberrations. Type of event as per color legend at the bottom right. *Of note, the depicted oncoprint is an example of the final version, accessible by request http://r2.amc.nl and publicly available after acceptance.*

(**b**) Type of detected events in the cohort.

(**c**) Five most frequently altered genes, grouped per pathway. Type of event as per color legend.

**Supplemental Figure 2. R2 Genomics Analysis and Visualization platform**

**Supplemental Figure 4. Potentially actionable somatic events, highlighted by cancer subtype**

(**a**) Frequency of actionable SNVs and InDels observed in 2 or more samples, highlighted by cancer subtype.

(**b**) Frequency of all gene fusions, highlighted by cancer subtype. Potentially actionable gene fusions are indicated by an asterisk (*).

(**c**) Frequency of actionable CNVs observed in 2 or more samples, highlighted by cancer subtype.

**Supplemental Figure 5.** tSNE plot of DNA methylation results of >70000 pediatric tumors, indicating clustering with neuroblastoma samples not rhabdoid tumors. (Kindly provided by INFORM).

**Supplemental Figure 6. Somatic events reported in consecutive samples indicate temporal heterogeneity**.

(**a**) Detected events in primary (*in* *brown*) or metastatic site (*in yellow*) of 12 patients, as obtained over time: alveolar rhabdomyosarcoma (aRMS, *n=*3), Ewing sarcoma (Ewing, *n=*3), osteosarcoma (OS, *n=*2), neuroblastoma (NBL, *n=*2), nasopharyngeal carcinoma (NPC, *n=*1), clear cell sarcoma (CCS, *n=*1). All samples except for P07 were obtained at consecutive timepoints. Three pairs without events are not depicted: two mesenchymal chondrosarcomas and one malignant rhabdoid tumor, respectively.

(**b**) Venn diagrams comparing somatic findings in primary (*in* *brown*) versus metastatic site (*in yellow*) in three patients. Samples of the patient with aRMS were obtained at the same timepoint; the NBL samples were sequenced at different time points.

**Supplemental Figure 7. Barriers to applying molecularly matched treatments**.

(**a**) In the subgroup of patients with 53 targets of very high, *high*, or *moderate* priority, molecularly matched treatment was applied in 24%. (**b**) Percentage of patients assigned to molecularly matched treatments, related to the target priority score as depicted on the x-axis. (**c**) Most relevant barriers to applying molecularly matched treatments, as indicated by treating physicians.

**Supplemental Table 1. Protocol details, in- and exclusion criteria of the iTHER studies.**

**Supplemental Table 2. Genes examined for germline variants.**

The gene panel was selected on the pediatric cancer predisposition syndrome gene panel, 2021 version 1.^1^

See also [pediatric-cancer-predisposition-genepanel.nl](https://www.pediatric-cancer-predisposition-genepanel.nl)

AD autosomal dominant; AR autosomal recessive; XLR X-linked recessive

^#^ only 3'end deletions; * paternal imprinting

**Supplemental Table 3. Samples with revised or refined diagnosis.**

**Supplemental Table 4. Specific details of germline pathogenic variants.**

**Supplemental Table 5.**  **Specific details of molecularly matched treatments applied.**
