# Supplementary material for: Implementation of paediatric precision oncology into clinical practice: The Individualized Therapies for Children with cancer program ‘iTHER’
Source: Eur J Cancer. 2022 Nov;175:311–25. doi: 10.1016/j.ejca.2022.09.001 (PMC9586161; doi:10.1016/j.ejca.2022.09.001)
Supplement: Multimedia component 2 [file mmc2.docx]

## Extended methods: the Princess Máxima Center Molecular Profiling pipeline

##### Whole Exome Sequencing

Whole exome sequencing (WES) libraries at the Princess Máxima Center were generated from 150 ng DNA using the Hyperexome (Roche) or MedExome (Roche) capture and similarly NovaSeq 6000 sequencing platform (Illumina) with a target coverage of 100x for normal samples and 200x for tumor. Data were processed as per the GATK 4.0 best practices workflow for variant calling, using a wdl and cromwell based workflow. Reads were aligned to GRCh38 using bwamem v0.7.13, and QC was performed using FastQC (0.11.5) and picardTools (2.20.1). Somatic variants were identified using Mutect2 from GATK v4.1 and annotated using Vep (v92). Likewise, CNVs were identified using GATK v4.1.

##### mRNA sequencing

mRNA sequencing (RNA-seq) libraries at the Princess Máxima Center were generated from 300 ng RNA using the KAPA RNA HyperPrep Kit with RiboErase (Roche) and sequenced with NovaSeq 6000 (2x150 bp) (Illumina) with a target of achieving at least 40 million unique reads.

Data were analyzed as previously described.^1^ In brief, data were aligned using Star (version 2.7.0f) to GRCh38 and gencode (version 29) and quality control performed using Fastqc (version 0.11.5) and Picard (version 2.20.1). Gene fusion events were identified using Star fusion (version 1.6.0). Variant calling on the RNA data was performed as per GATK 4.0 best practices workflow (https://gatk.broadinstitute.org/hc/en-us/sections/360007226651-Best-Practices-Workflows). And finally, expression counts were calculated using Subread Counts.

##### Germline analysis

Reads were aligned to genome build GRCh38 using BWA.^2^ Calling of SNVs and InDel variants was performed using Genome Analysis Tool Kit (GATK)^3^ implemented according to the GATK best practices guidelines.^4^ Subsequently, all variants were annotated using Ensembl VEP version 92^5^ and converted to tabular output format using a custom build script. CNV-calling was performed following GATK best practices using an in-house compiled panel of normals. Afterwards the called segments were annotated and filtered using a custom R transcript for further interpretation. To identify germline variants with a high likelihood of being relevant in pediatric cancer development, we used a list of 139 genes with an evidence-based causal relation with pediatric cancer *(**Supplemental Table 2)*. Selection criteria for assembling this gene panel are described by Byrjalsen and colleagues^6^ and can be found on [pediatric-cancer-predisposition-genepanel.nl](https://www.pediatric-cancer-predisposition-genepanel.nl). Germline variants were analyzed in a stepwise manner to eventually classify them into five categories according to ACMG guidelines^7^: pathogenic (class 5), likely pathogenic (class 4), variant of unknown significance (class 3), likely benign (class 2), and benign (class 1). First, we filtered for high quality variants, determined by mapping quality (≥30), read depth (≥10), and variant allele frequency (VAF, ≥0.25). Second, we selected exonic variants and intronic variants up to 20 nucleotides from the exon-intron junction with a minor allele frequency (MAF) of ≤1% in the gnomAD public variant database^8^ and prioritized variants based on variant annotation pathogenicity scores. The functional effect of missense variants was predicted by the *in silico* tools CADD (v1.5), SIFT (v5.2.2), Align-GVGD and Polyphen-2 (v2.2.2)]. We used Alamut Visual (v2.15.0, https://www.interactive-biosoftware.com/alamut-visual/) to evaluate the effect of variants on splicing. Variants of interest included: 1) truncating variants in tumor suppressor genes, 2) missense variants with a CADD score >15 and at least two *in silico* protein prediction tools predicting a possible functional effect, and 3) variants that likely affect splicing. Finally, we checked whether the variants of interest were already reported in public databases, including Clinvar, Leiden Open Variation Database (LOVD), International Agency for Research on Cancer *TP53* (IARC *TP53*), Human Gene Mutation Database (HGMD) and Catalogue of Somatic Mutations in Cancer (COSMIC). Class 4 and 5 variants (collectively referred to as P/LP variants) were manually assessed using Integrative Genomic Viewer (IGV, v2.8.12) to confirm their validity in sequence data from both normal and tumor and, subsequently, reviewed by the Multidisciplinary Tumor Board (MTB). In addition, we analyzed the tumor sequence data for second hit pathogenic variants and loss of heterozygosity (LOH). P/LP variants in genes that cause autosomal dominant (AD) inherited cancer predisposition syndromes (CPSs), and biallelic variants in genes that cause autosomal recessive (AR) inherited CPSs were reported. Monoallelic pathogenic variants in genes purely causing AR inherited conditions were only reported if the parents of the child were consanguineous. Based on available literature and tumor WES data we assessed the likelihood of causality of the germline variants for the type of cancer diagnosed in these children. Variants were categorized into two groups: ‘causative’ and ‘uncertain causality’. The causative group only includes variants in genes known to predispose to the cancer type that was diagnosed in the patient. P/LP variants in pediatric cancer predisposing genes that, based on the current available literature, are not associated with the type of cancer in these children were classified as uncertain causality’.

**References**

1 Hehir-Kwa, J. Y. *et al.* Improved Gene Fusion Detection in Childhood Cancer Diagnostics Using RNA Sequencing. *JCO Precis Oncol* **6**, e2000504, doi:10.1200/po.20.00504 (2022).

2 Li, H. & Durbin, R. Fast and accurate short read alignment with Burrows–Wheeler transform. *Bioinformatics* **25**, 1754-1760, doi:10.1093/bioinformatics/btp324 (2009).

3 McKenna, A. *et al.* The Genome Analysis Toolkit: a MapReduce framework for analyzing next-generation DNA sequencing data. *Genome Res* **20**, 1297-1303, doi:10.1101/gr.107524.110 (2010).

4 Van der Auwera, G. A. *et al.* From FastQ Data to High-Confidence Variant Calls: The Genome Analysis Toolkit Best Practices Pipeline. *Current Protocols in Bioinformatics* **43**, 11.10.11-11.10.33, doi:https://doi.org/10.1002/0471250953.bi1110s43 (2013).

5 McLaren, W. *et al.* The Ensembl Variant Effect Predictor. *Genome Biology* **17**, 122, doi:10.1186/s13059-016-0974-4 (2016).

6 Byrjalsen, A. *et al.* Selection criteria for assembling a pediatric cancer predisposition syndrome gene panel. *Fam Cancer* **20**, 279-287, doi:10.1007/s10689-021-00254-0 (2021).

7 Richards, S. *et al.* Standards and guidelines for the interpretation of sequence variants: a joint consensus recommendation of the American College of Medical Genetics and Genomics and the Association for Molecular Pathology. *Genet Med* **17**, 405-424, doi:10.1038/gim.2015.30 (2015).

8 Zhang, J. *et al.* Germline Mutations in Predisposition Genes in Pediatric Cancer. *N Engl J Med* **373**, 2336-2346, doi:10.1056/NEJMoa1508054 (2015).
