# Supplementary material for: Implementation of paediatric precision oncology into clinical practice: The Individualized Therapies for Children with cancer program ‘iTHER’
Source: Eur J Cancer. 2022 Nov;175:311–25. doi: 10.1016/j.ejca.2022.09.001 (PMC9586161; doi:10.1016/j.ejca.2022.09.001)
Supplement: Multimedia component 3 [file mmc3.docx]

# ­SUPPLEMENTAL FILES

| **Individualized Therapies (iTHER) for children 1.0** |
| --- |
| Inclusion criteria |
| - Relapsed/refractory pediatric cancer, established by standard diagnostic methods. - No available standard treatment or study protocol. - Life-expectancy of at least 10 weeks. - Written informed consent according to local law and legislation. - Age <30 years. |
| Exclusion criteria |
| - Biopsy considered unsafe. - Severe organ toxicity precluding undergoing any of the procedures mentioned in this protocol. - Any other condition that may hamper participation according to the treating physician. |
| End of follow up: April 1^st^, 2021 |
| ID: NL56826.078.16; Netherlands Trial Register Trial NL5728 (NTR5915) |
| Sponsor: Dutch Childhood Oncology Group |
|  |
| **Individualized Therapies (iTHER) for children 2.0** |
| Inclusion criteria |
| - Relapsed/refractory pediatric cancer, which was established by standard diagnostic   methods OR initially diagnosed pediatric cancer patients for whom no standard treatment  strategy is available or who have a dismal outcome with current treatment protocols.   - A tissue sample or tumor DNA/RNA as well as normal DNA was obtained as standard of care at diagnosis, resection or at the event of refractory or relapsed disease or is planned with the purpose to confirm the diagnosis or a suspected relapse/refractory disease. - Life-expectancy of at least 12 weeks. - Written informed consent according to local law and legislation - Age <30 years. |
| Exclusion criteria |
| - If patients and parents do not want to be informed about detected germline abnormalities in childhood cancer-associated genes. |
| ID: METC-Protocol number: 20/150/C |
| Sponsor: Princess Máxima Center for pediatric oncology |

**Supplemental Table 1. Protocol details, in- and exclusion criteria of the iTHER studies.**
