# Supplementary material for: Implementation of paediatric precision oncology into clinical practice: The Individualized Therapies for Children with cancer program ‘iTHER’
Source: Eur J Cancer. 2022 Nov;175:311–25. doi: 10.1016/j.ejca.2022.09.001 (PMC9586161; doi:10.1016/j.ejca.2022.09.001)
Supplement: Multimedia component 4 [file mmc4.docx]

# ­SUPPLEMENTAL FILES _ TABLE2

| **Gene** | **Inheritance** |
| --- | --- |
| *A2ML1* | AD |
| *ABCB11* | AR |
| *ACD* | AD/AR |
| *AIP* | AD |
| *ALK* | AD |
| *APC* | AD |
| *ATM* | AD/AR |
| *BAP1* | AD |
| *BLM* | AR |
| *BRAF* | AD |
| *BRCA2* | AD/AR |
| *BRIP1* | AD/AR |
| *BUB1B* | AR |
| *CBL* | AD |
| *CD27* | AR |
| *CD70* | AR |
| *CDC73* | AD |
| *CDH1* | AD |
| *CDKN1C* | AD |
| *CDKN2A* | AD |
| *CEBPA* | AD |
| *CEP57* | AR |
| *CREBBP* | AD |
| *CTC1* | AR |
| *CTLA4* | AD |
| *CTR9* | AD |
| *DDB2* | AR |
| *DICER1* | AD |
| *DIS3L2* | AR |
| *DKC1* | XLR |
| *EGLN1* | AD |
| *EGLN2* | AD |
| *ELP1* | AD |
| *EPAS1* | AD |
| *EPCAM ^#^* | AD |

**Supplemental Table 2. Genes examined for germline variants.**

The gene panel was selected on the pediatric cancer predisposition syndrome gene panel, 2021 version 1.^1^

See also [pediatric-cancer-predisposition-genepanel.nl](https://www.pediatric-cancer-predisposition-genepanel.nl)

AD autosomal dominant; AR autosomal recessive; XLR X-linked recessive

^#^ only 3'end deletions; * paternal imprinting

| **Gene** | **Inheritance** |
| --- | --- |
| *ERCC2* | AR |
| *ERCC3* | AR |
| *ERCC4* | AR |
| *ERCC5* | AR |
| *ETV6* | AD |
| *EZH2* | AD |
| *FANCA* | AR |
| *FANCB* | XLR |
| *FANCC* | AR |
| *FANCD2* | AR |
| *FANCE* | AR |
| *FANCF* | AR |
| *FANCG* | AR |
| *FANCI* | AR |
| *FANCL* | AR |
| *FAS* | AD |
| *FBXW7* | AD |
| *FH* | AD |
| *GATA2* | AD |
| *GPC3* | XLR |
| *GPR161* | AD |
| *HAVCR2* | AR |
| *HRAS* | AD |
| *IKZF1* | AD |
| *ITK* | AR |
| *KRAS* | AD |
| *LIG4* | AR |
| *LZTR1* | AD/AR |
| *MAP2K1* | AD |
| *MAP2K2* | AD |
| *MDH2* | AD |
| *MEN1* | AD |
| *MLH1* | AD/AR |
| *MSH2* | AD/AR |
| *MSH6* | AD/AR |

| **Gene** | **Inheritance** |
| --- | --- |
| *NBN* | AR |
| *NF1* | AD |
| *NF2* | AD |
| *NHP2* | AR |
| *NOP10* | AR |
| *NRAS* | AD |
| *NSD1* | AD |
| *PALB2* | AD/AR |
| *PARN* | AR |
| *PAX5* | AD |
| *PHOX2B* | AD |
| *PIK3CA* | AD |
| *PMS2* | AD/AR |
| *POLH* | AR |
| *PTCH1* | AD |
| *PTCH2* | AD |
| *PTEN* | AD |
| *PTPN11* | AD |
| *RAF1* | AD |
| *RB1* | AD |
| *RECQL4* | AR |
| *REST* | AD |
| *RET* | AD |
| *RIT1* | AD |
| *RMRP* | AR |
| *RPL11* | AD |
| *RPL35A* | AD |
| *RPL5* | AD |
| *RPS10* | AD |
| *RPS17* | AD |
| *RPS19* | AD |
| *RPS24* | AD |
| *RPS26* | AD |
| *RPS27* | AD |
| *RTEL1* | AD/AR |

| **Gene** | **Inheritance** |
| --- | --- |
| *RUNX1* | AD |
| *SAMD9* | AD |
| *SAMD9L* | AD |
| *SBDS* | AR |
| *SDHA* | AD |
| *SDHAF2** | AD |
| *SDHB* | AD |
| *SDHC* | AD |
| *SDHD** | AD |
| *SETBP1* | AD |
| *SH2D1A* | XLR |
| *SHOC2* | AD |
| *SMARCA4* | AD |
| *SMARCB1* | AD |
| *SMARCE1* | AD |
| *SOS1* | AD |
| *STK11* | AD |
| *SUFU* | AD/AR |
| *TERC* | AD |
| *TERT* | AD/AR |
| *TINF2* | AD |
| *TP53* | AD |
| *TRIM28* | AD |
| *TRIM37* | AR |
| *TRIP13* | AR |
| *TSC1* | AD |
| *TSC2* | AD |
| *USB1* | AR |
| *VHL* | AD |
| *WAS* | XLR |
| *WRAP53* | AR |
| *WT1* | AD |
| *XPA* | AR |
| *XPC* | AR |

References

1 Byrjalsen, A. *et al.* Selection criteria for assembling a pediatric cancer predisposition syndrome gene panel. *Fam Cancer* **20**, 279-287, doi:10.1007/s10689-021-00254-0 (2021).
