# Supplementary material for: Implementation of paediatric precision oncology into clinical practice: The Individualized Therapies for Children with cancer program ‘iTHER’
Source: Eur J Cancer. 2022 Nov;175:311–25. doi: 10.1016/j.ejca.2022.09.001 (PMC9586161; doi:10.1016/j.ejca.2022.09.001)
Supplement: Multimedia component 5 [file mmc5.docx]

# ­SUPPLEMENTAL FILES_TABLE3

| **Histologic diagnosis at registration** | **Revised/refined molecular diagnosis** | **Method** |
| --- | --- | --- |
| Embryonal rhabdomyosarcoma | *PAX3-FOXO1* fusion positive alveolar rhabdomyosarcoma | RNA-seq |
| Embryonal rhabdomyosarcoma | Novel *PAX3-WWTR1* fusion | RNA-seq |
| Undifferentiated sarcoma | *BCOR-CCNB3* fusion positive sarcoma | RNA-seq |
| Malignant peripheral nerve sheath tumor | *TPM3-NTRK1* fusion driven MPNST | RNA-seq |
| Malignant Rhabdoid Tumor | Neuroblastoma with *SMARCA4* L1525P + loss of heterozygosity 19p | WES;  DNA methylation  *(as depicted in Suppl Fig 4)* |
| Atypical Teratoid Rhabdoid Tumor | High-grade glioma high-grade glioma, H3-wildtype and IDH-wildtype; secondary malignancy | WES;  DNA methylation |
| Low-Grade Glioma | Pilocytic astrocytoma | DNA methylation^#^ |
| Medulloblastoma, group 4 | Medulloblastoma, group 3/4 subtype VII | DNA methylation^#^ |

**Supplemental Table 3. Samples with revised or refined diagnosis.**
