# Supplementary material for: Implementation of paediatric precision oncology into clinical practice: The Individualized Therapies for Children with cancer program ‘iTHER’
Source: Eur J Cancer. 2022 Nov;175:311–25. doi: 10.1016/j.ejca.2022.09.001 (PMC9586161; doi:10.1016/j.ejca.2022.09.001)
Supplement: Multimedia component 7 [file mmc7.docx]

# ­SUPPLEMENTAL FILES_TABLE5

| **Diagnosis** | **Genes targeted** | **Alteration** | **Priority** | **Targeted therapy received** | **Therapy**  **duration (days)** |
| --- | --- | --- | --- | --- | --- |
| Acute myeloid leukemia | *FGR;*  *LYN* | overexpression;  overexpression | 4 = intermediate | dasatinib & cytarabine | 9 |
| High-grade glioma | *PTEN* | SNV | 3 = moderate | everolimus | 21 |
| High-grade glioma | *PARP1* | overexpression | 4 = intermediate | olaparib & irinotecan | 30 |
| Neuroblastoma | *ALK* | amplification and  overexpression | 1 = very high | crizotinib | 32 |
| Neuroblastoma | *ALK* | SNV | 1 = very high | crizotinib & temsirolimus | 32 |
| Rhabdomyosarcoma | *CDKN2A* | biallelic deletion | 3 = moderate | ribociclib & topotecan & temozolomide | 37 |
| Acute myeloid leukemia | *EZH2* | SNV | 4 = intermediate | bortezomib & cytarabine & HSCT | 38 |
| Neuroblastoma | *CDKN2A* | deletion | 3 = moderate | ribociclib | 48 |
| Neuroblastoma | *CDKN2A* | deletion | 3 = moderate | ribociclib | 49 |
| Acute lymphoblastic leukemia | *NRAS* | SNV | 2 = high | selumetinib & dexamethasone | 55 |
| Medulloblastoma | *CDK6* | amplification | 1 = very high | ribociclib & topotecan & temozolomide | 56 |
| Rhabdomyosarcoma | *ALK* | gain and  high expression | 6 = low | crizotinib | 58 |
| High-grade glioma | NA | hypermutator | 2 = high | nivolumab & entinostat | 72 |
| Neuroblastoma | *HGF* | overexpression | 4 = intermediate | crizotinib | 73 |
| Rhabdomyosarcoma | *FGFR4* | overexpression | 4 = intermediate | pazopanib & topotecan | 31 |
| Rhabdomyosarcoma | *CDK4* | amplification | 2 = high | ribociclib & topotecan & temozolomide | 89 |
| Neuroblastoma | *ALK;  mTOR* | SNV;  overexpression | 1 = very high | crizotinib & temsirolimus | 28 |
| Neuroblastoma | *BCL2* | high expression | 6 = low | venetoclax & cyclophosphamide & topotecan | 99 |
| Rhabdomyosarcoma | *ATM* | InDel | 3 = moderate | olaparib & irinotecan | 140 |
| Ewing sarcoma | *CCND1* | high expression | 6 = low | ribociclib & topotecan & temozolomide | 176 |
| Ewing sarcoma | *CCND1* | high expression | 6 = low | ribociclib & topotecan & temozolomide | 181 |

**Supplemental Table 5.**  **Specific details of molecularly matched treatments applied.**
