# Supplementary material for: Implementation of paediatric precision oncology into clinical practice: The Individualized Therapies for Children with cancer program ‘iTHER’
Source: Eur J Cancer. 2022 Nov;175:311–25. doi: 10.1016/j.ejca.2022.09.001 (PMC9586161; doi:10.1016/j.ejca.2022.09.001)
Supplement: Multimedia component 8 [file mmc8.pdf]

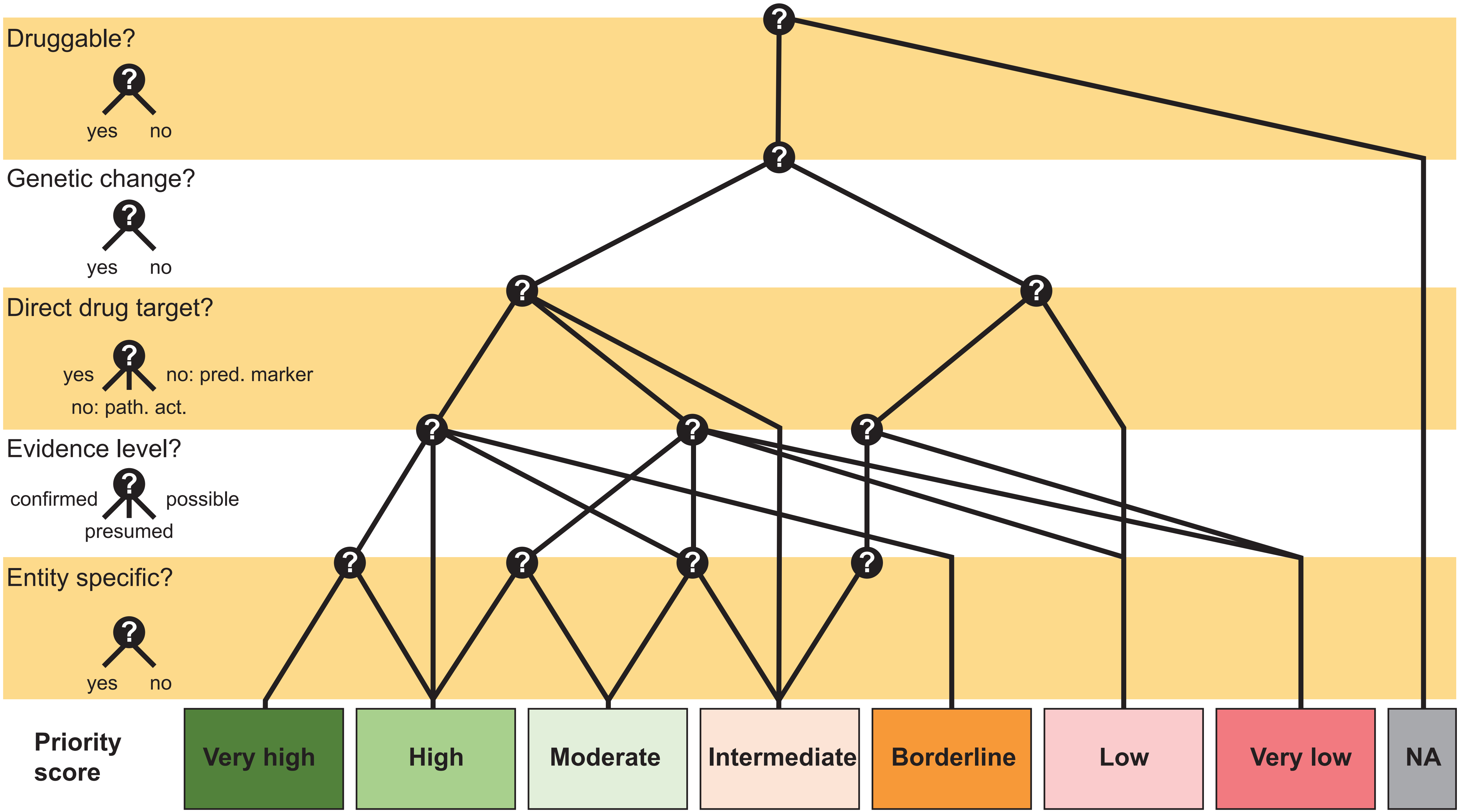

Druggable?
yes
no
Genetic change?
yes
no
Direct drug target?
yes
no: pred. marker
no: path. act.
Evidence level?
confirmed
possible
presumed
Entity specific?
yes
no
Priority score
Very high
High
Moderate
Intermediate
Borderline
Low
Very low
NA
