# Supplementary material for: Implementation of paediatric precision oncology into clinical practice: The Individualized Therapies for Children with cancer program ‘iTHER’
Source: Eur J Cancer. 2022 Nov;175:311–25. doi: 10.1016/j.ejca.2022.09.001 (PMC9586161; doi:10.1016/j.ejca.2022.09.001)
Supplement: Multimedia component 9 [file mmc9.pdf]

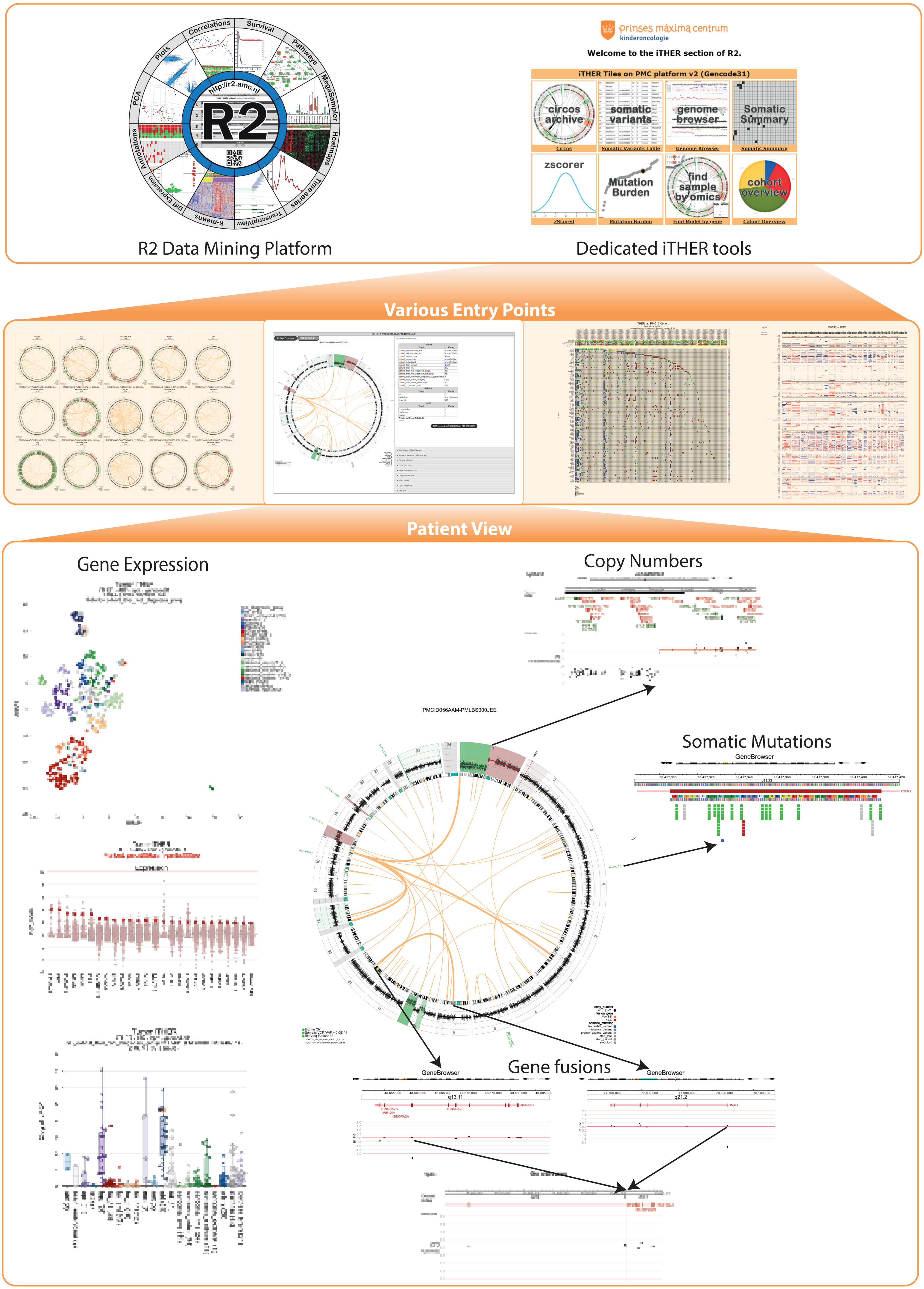

R2 Data Mining Platform
Correlations
Survival
Pathways
MegaSampler
Heatmaps
Time series
TranscriptView
K-means
Diff Expression
Annotations
PCA
Plots
http://r2.amc.nl
R2
prinses máxima centrum
kinderoncologie
Welcome to the iTHER section of R2.
iTHER Tiles on PMC platform v2 (Gencode31)
circos archive
Circos
somatic variants
Somatic Variants Table
genome browser
Genome Browser
Somatic Summary
Somatic Summary
zscorer
ZScored
Mutation Burden
Mutation Burden
find sample by omics
Find Model by gene
cohort overview
Cohort Overview
Dedicated iTHER tools
Various Entry Points
Patient View
Gene Expression
Copy Numbers
PMCID056AAM-PMLBS000JEE
Somatic Mutations
GeneBrowser
Gene fusions
GeneBrowser
GeneBrowser
