# Supplementary figures and images for: Implementation of paediatric precision oncology into clinical practice: The Individualized Therapies for Children with cancer program ‘iTHER’
Source: Eur J Cancer. 2022 Nov;175:311–25. doi: 10.1016/j.ejca.2022.09.001 (PMC9586161; doi:10.1016/j.ejca.2022.09.001)

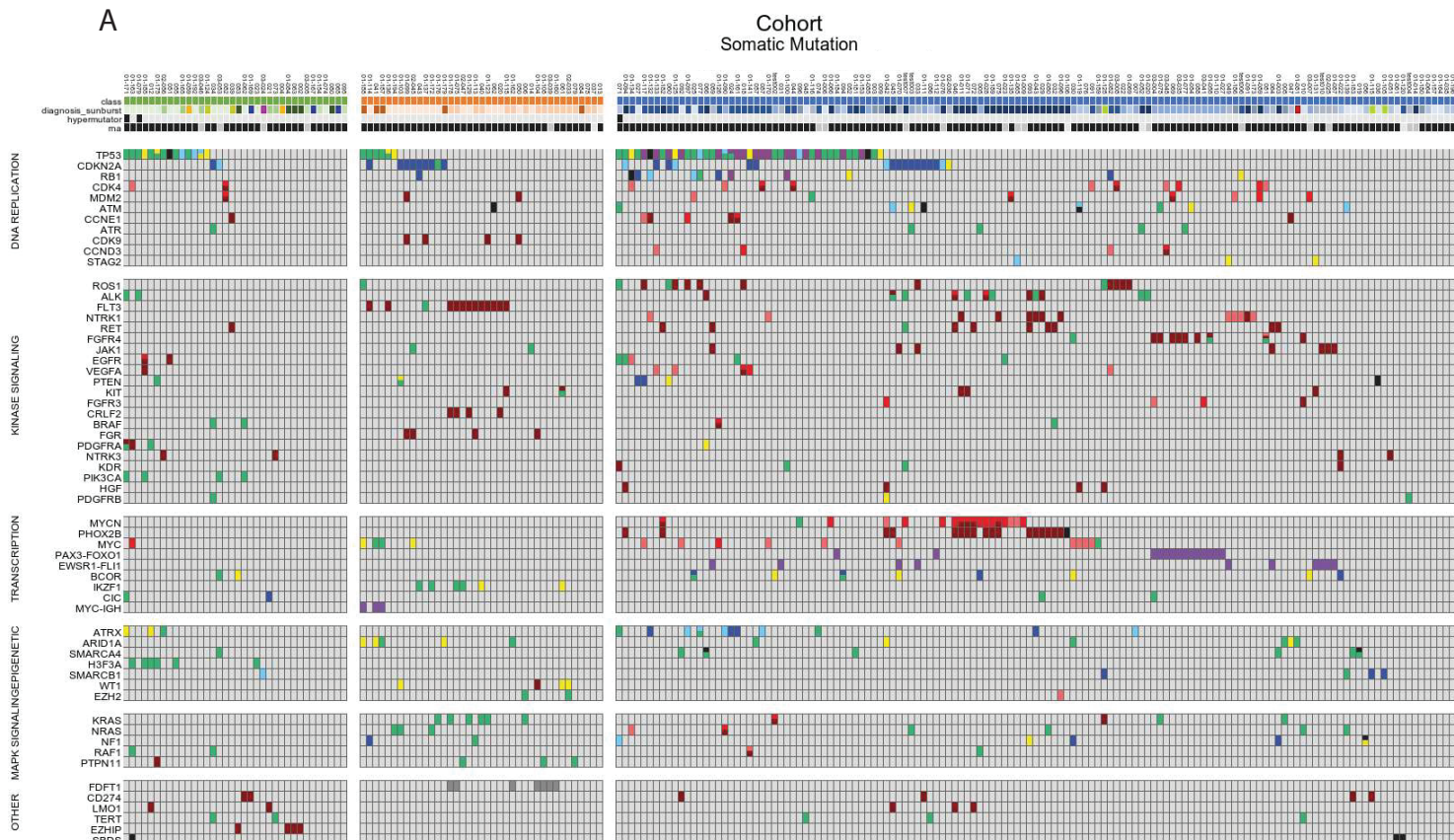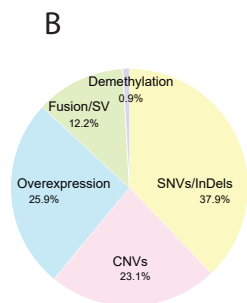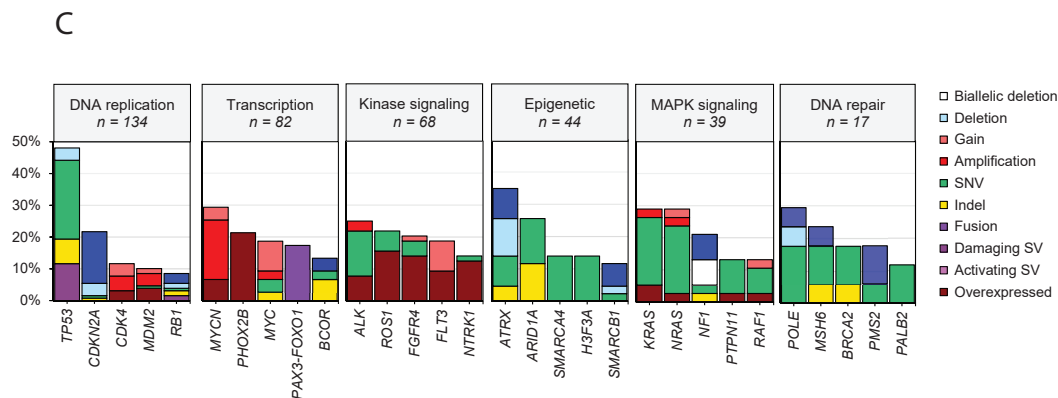

Supplement: Multimedia component 10 [file mmc10.pdf]

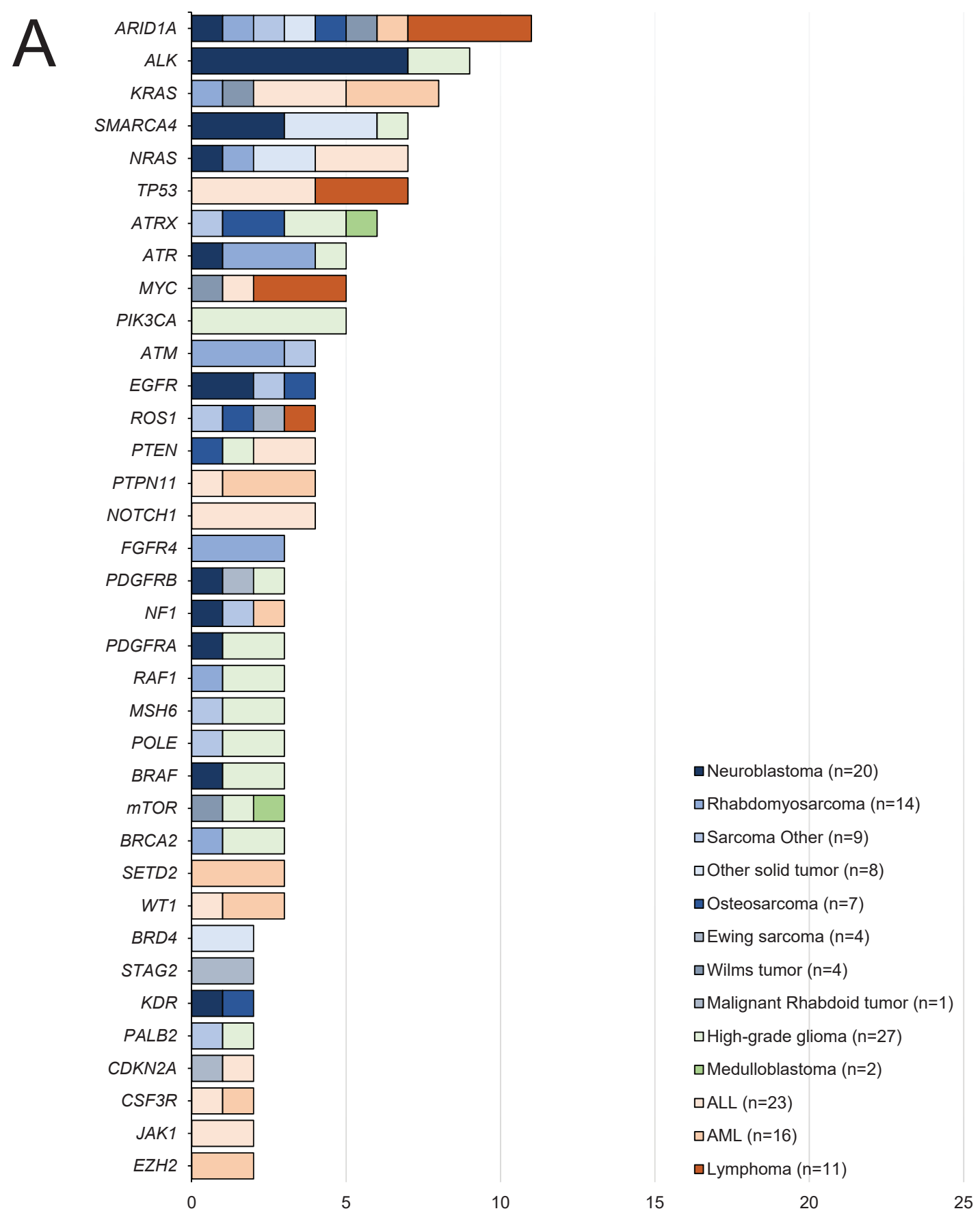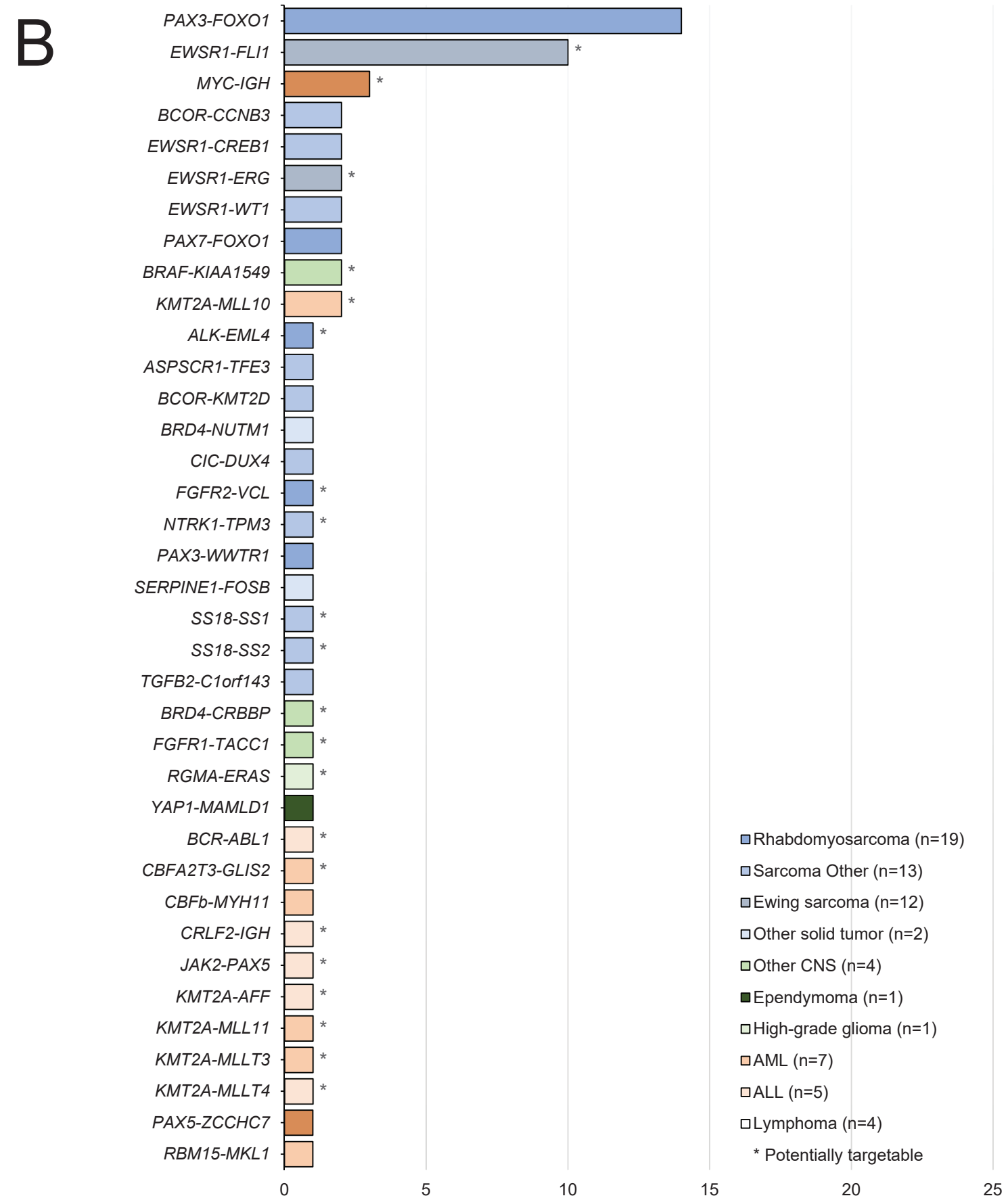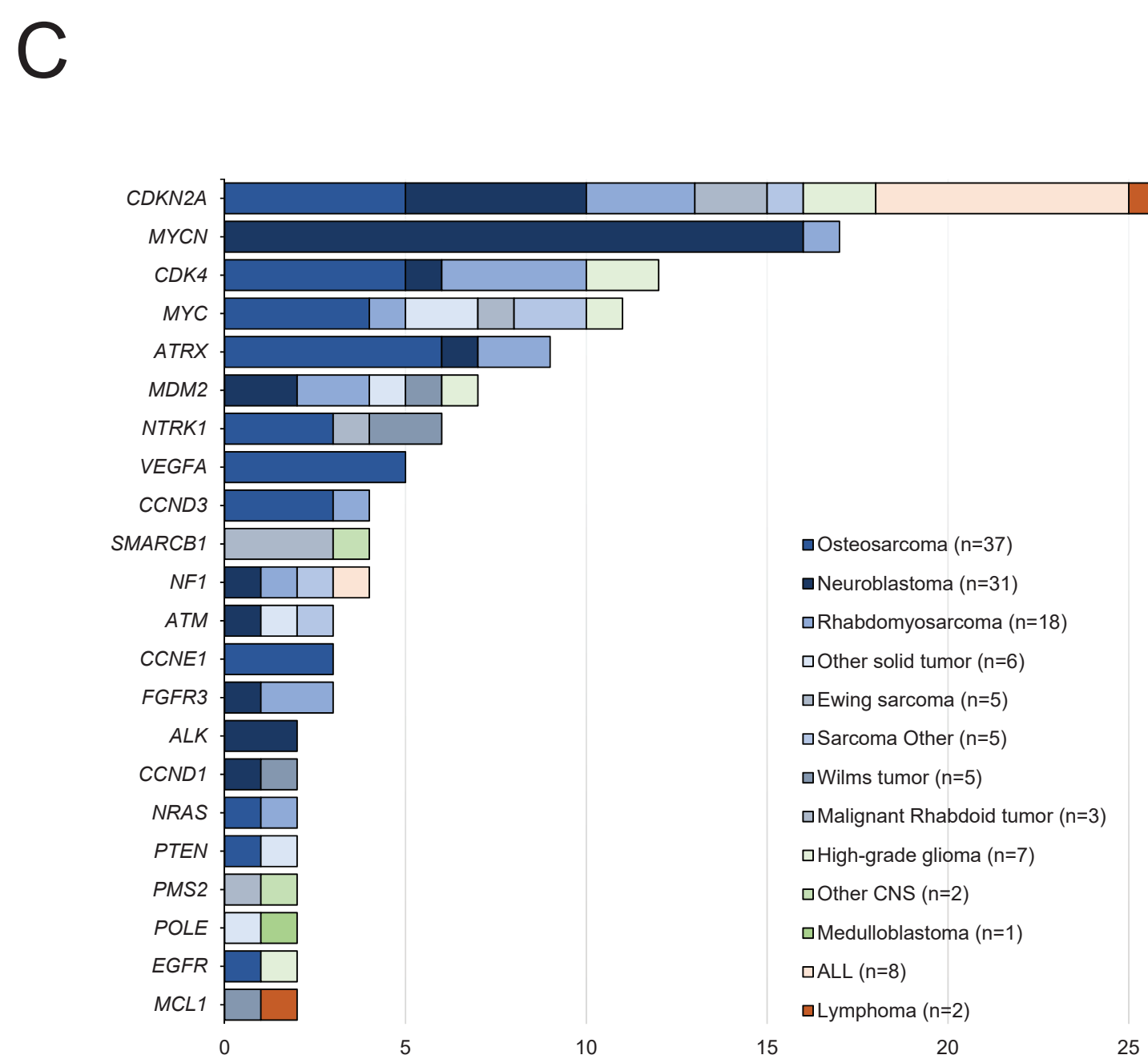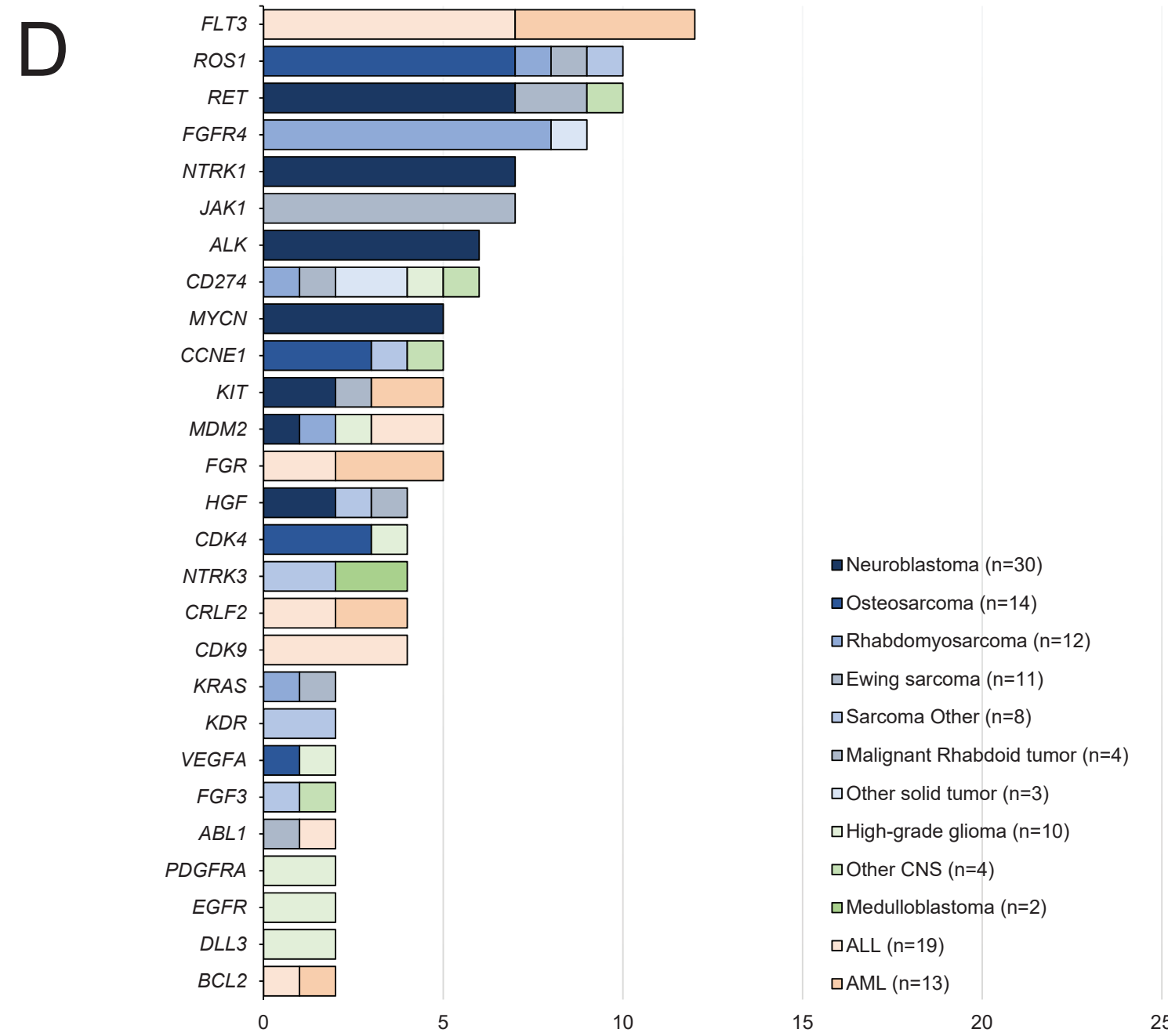

Supplement: Multimedia component 11 [file mmc11.pdf]

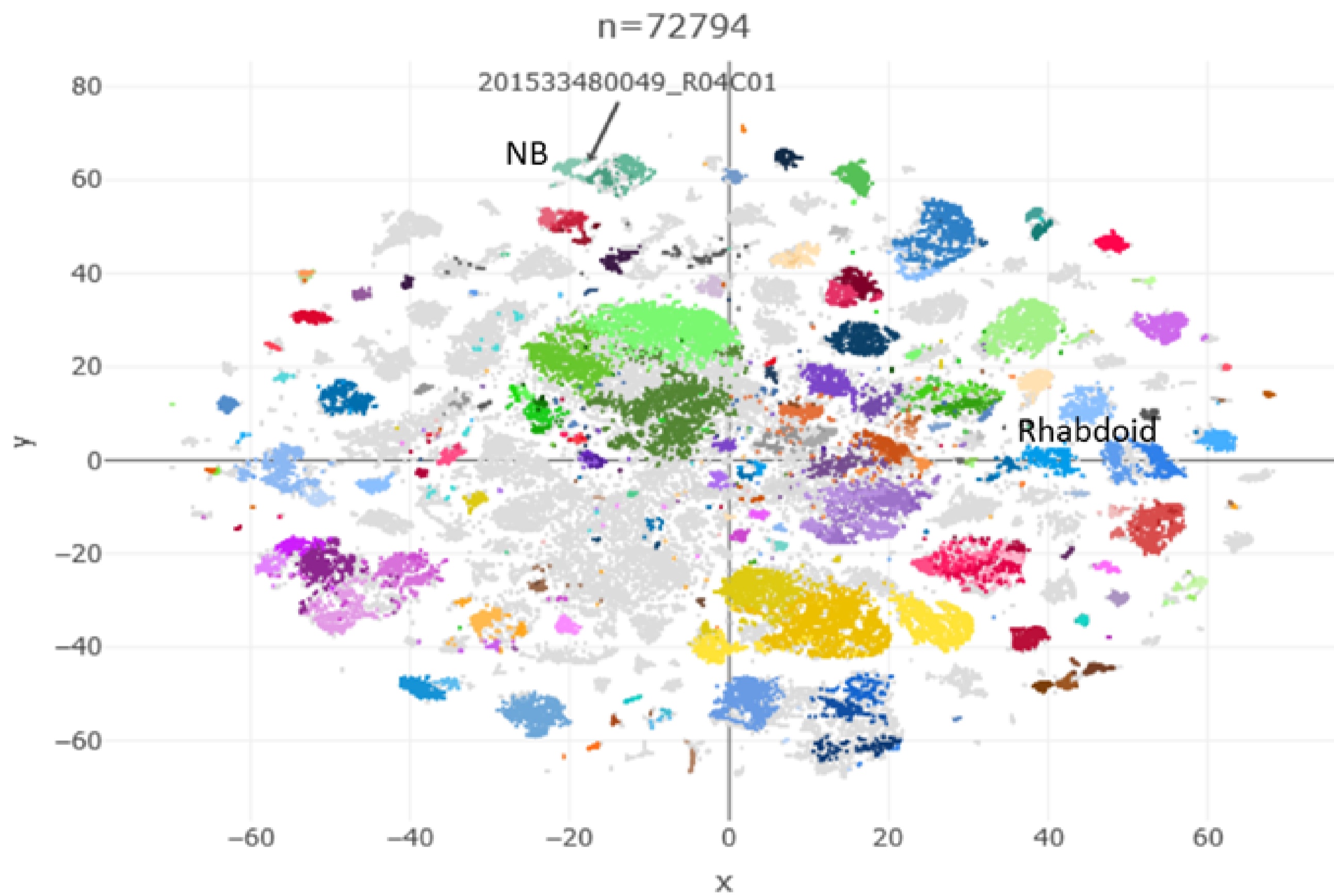

Supplement: Multimedia component 12 [file mmc12.pdf]

A

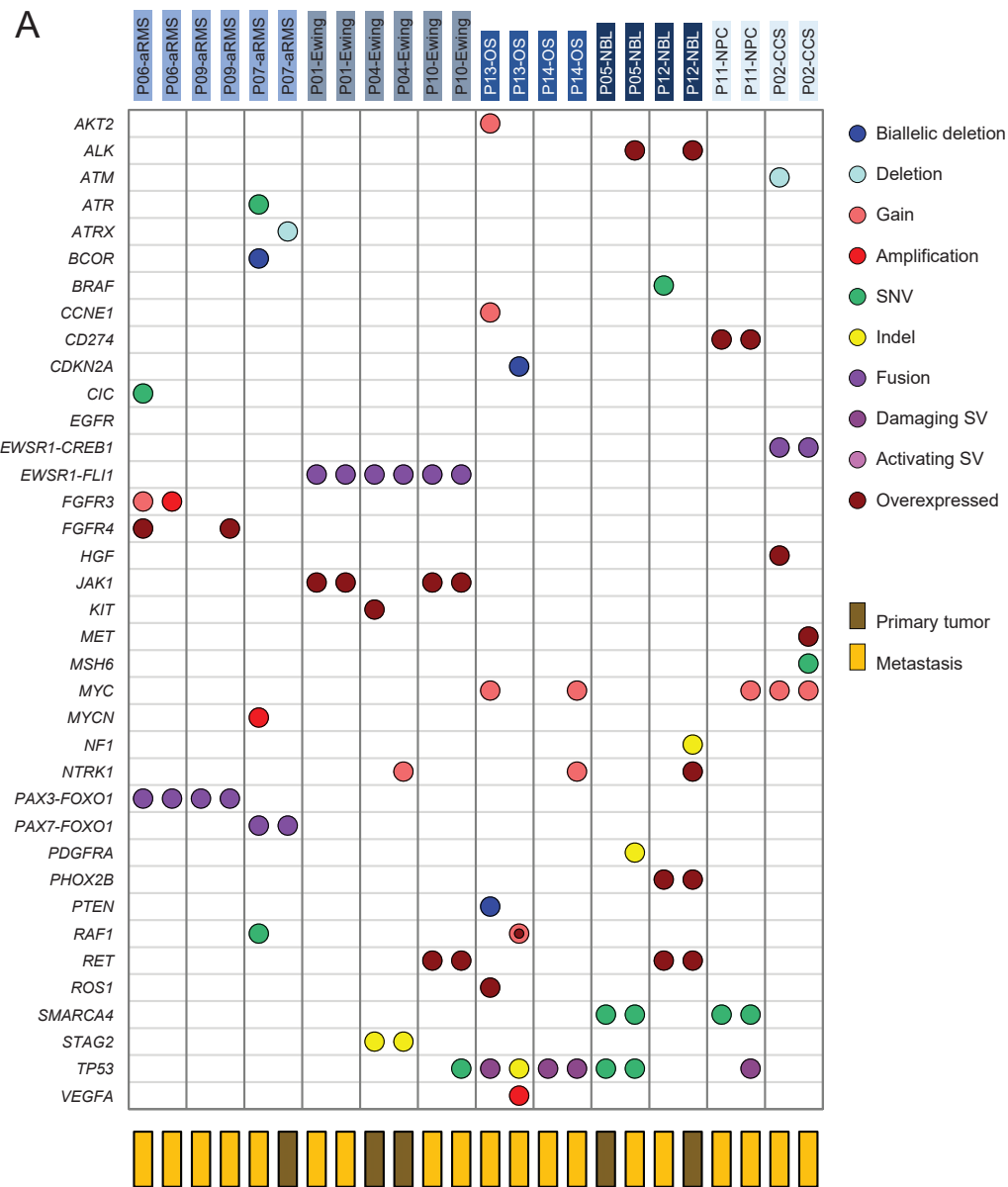

B

P07-aRMS

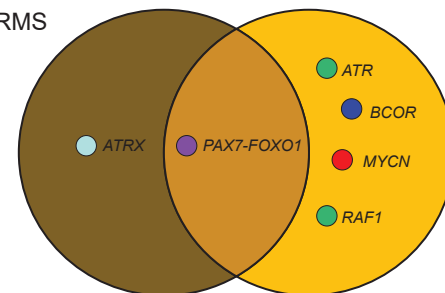

P05-NBL

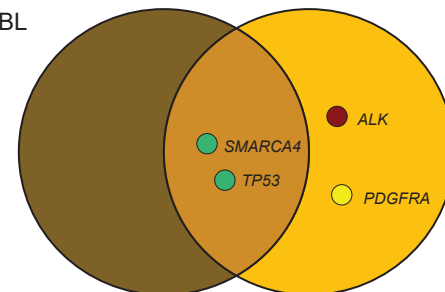

P12-NBL

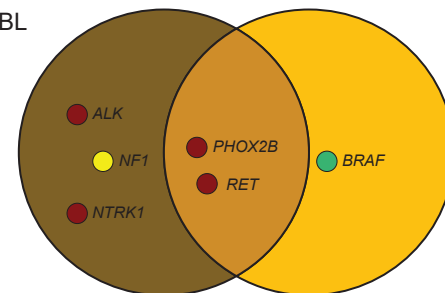

Supplement: Multimedia component 13 [file mmc13.pdf]
