# Supplementary material for: Implementation of paediatric precision oncology into clinical practice: The Individualized Therapies for Children with cancer program ‘iTHER’
Source: Eur J Cancer. 2022 Nov;175:311–25. doi: 10.1016/j.ejca.2022.09.001 (PMC9586161; doi:10.1016/j.ejca.2022.09.001)
Supplement: Multimedia component 14 [file mmc14.pdf]

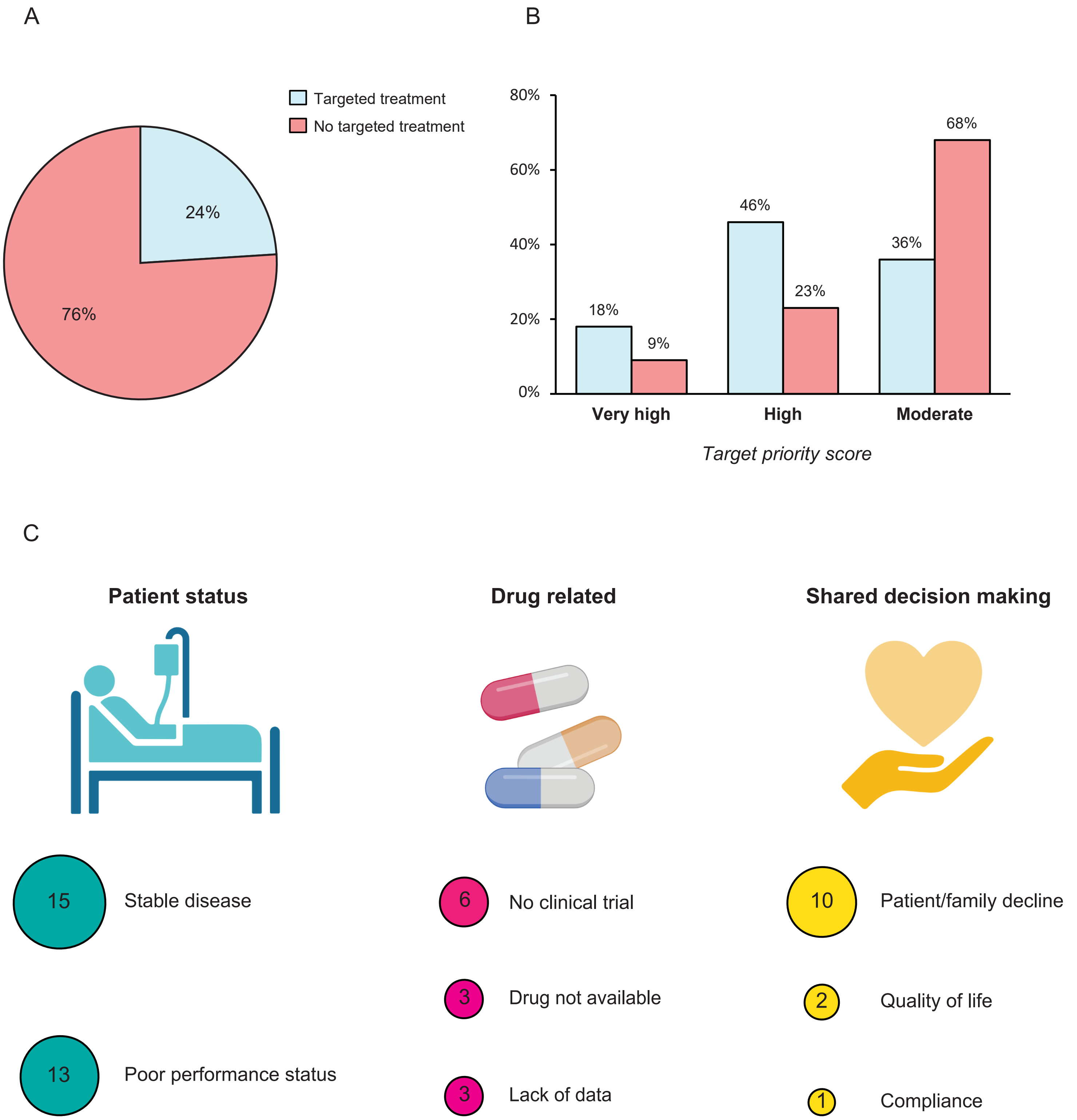

A
Targeted treatment
No targeted treatment
24%
76%
B
80%
60%
40%
20%
0%
18%
9%
46%
23%
36%
68%
Very high
High
Moderate
Target priority score
C
Patient status
Drug related
Shared decision making
15
Stable disease
13
Poor performance status
6
No clinical trial
3
Drug not available
3
Lack of data
10
Patient/family decline
2
Quality of life
1
Compliance
